# Supplementary material for: Spatially explicit analysis identifies significant potential for bioenergy with carbon capture and storage in China
Source: Nat Commun. 2021 May 26;12:3159. doi: 10.1038/s41467-021-23282-x (PMC8154910; doi:10.1038/s41467-021-23282-x)
Supplement: Supplementary file 1 — Supplementary Information [file 41467_2021_23282_MOESM1_ESM.pdf]

Supplementary Materials for

Spatially explicit analysis identifies significant potential for bioenergy with carbon capture and storage in China

Xiaofan Xing[1], Rong Wang[1,2,3,4,5,6*], Nico Bauer[7], Philippe Ciais[8], Junji Cao[9], Jianmin Chen[1,2,3], Xu Tang[1,2,3], Lin Wang[1], Xin Yang[1], Olivier Boucher[10], Daniel Goll[11], Josep Peñuelas[12,13], Ivan A. Janssens[14], Yves Balkanski[8], James Clark[15], Jianmin Ma[16], Bo Pan[17], Shicheng Zhang[1], Xingnan Ye[1], Yutao Wang[1], Qing Li[1], Gang Luo[1], Guofeng Shen[16], Wei Li[18], Yechen Yang[1], Siqing Xu[1]

[1]Shanghai Key Laboratory of Atmospheric Particle Pollution and Prevention, Department of Environmental Science and Engineering, Fudan University, Shanghai 200438, China.

[2]IRDR International Center of Excellence on Risk Interconnectivity and Governance on Weather/Climate Extremes Impact and Public Health (WECEIPHE), Fudan University, Shanghai 200438, China.

[3]Institute of Atmospheric Sciences, Fudan University, Shanghai 200438, China.

[4]Center for Urban Eco-Planning & Design, Fudan University, Shanghai 200438, China.

[5]Big Data Institute for Carbon Emission and Environmental Pollution, Fudan University, Shanghai 200438, China.

[6]Shanghai Institute of Pollution Control and Ecological Security, Shanghai 200092, China.

[7]Potsdam Institute for Climate Impact Research (PIK), Member of the Leibniz Association, Potsdam 14412, Germany.

[8]Laboratoire des Sciences du Climat et de l'Environnement, CEA CNRS UVSQ, Gif-sur-Yvette 91190, France.

[9]Institute of Atmospheric Physics, Chinese Academy of Sciences, Beijing 100029, China.

[10]Institut Pierre-Simon Laplace, Sorbonne Université / CNRS, Paris 75252, France.

[11]Lehrstuhl für Physische Geographie mit Schwerpunkt Klimaforschung, Universität Augsburg, Augsburg, Germany.

[12]CREAF, Cerdanyola del Vallès, Catalonia 08193, Spain.

[13]CSIC, Global Ecology Unit CREAF-CSIC-UAB, Bellaterra, Catalonia 08193, Spain.

[14]Department of Biology, University of Antwerp, Universiteitsplein 1, Wilrijk B 2610, Belgium.

[15]The University of York, Department of Chemistry, Green Chemistry Centre of Excellence, York YO10 5DD, UK.

[16]College of Urban and Environmental Sciences, Laboratory for Earth Surface Processes, Peking University, Beijing 100871, China.

[17]Environmental Science & Engineering, Kunming University of Science & Technology, Kunming 650500, China.

[18]Department of Earth System Science, Tsinghua University, Beijing 100871, China.

[*]Correspondence: Rong Wang (rongwang@fudan.edu.cn)

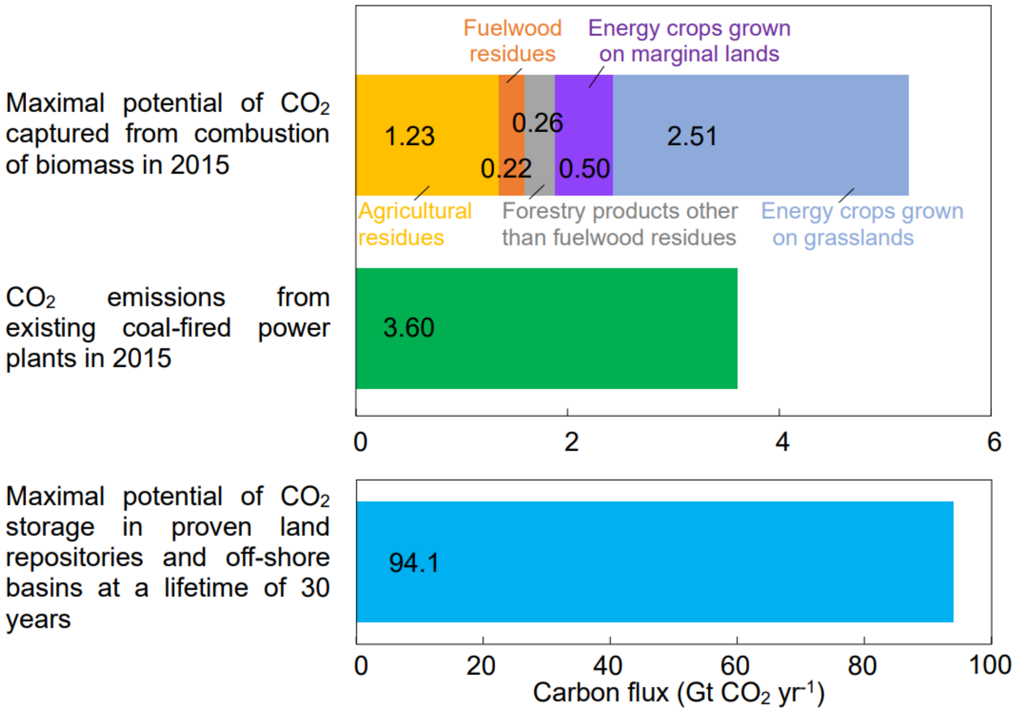

Figure S1. Carbon budget of BECCS in China. Maximal potential of CO_2 captured from combustion of biomass, CO_2 emissions from existing coal-fired power plants, and allowable annual CO_2 flux in the proven land repositories and off-shore basins are shown for year 2015.

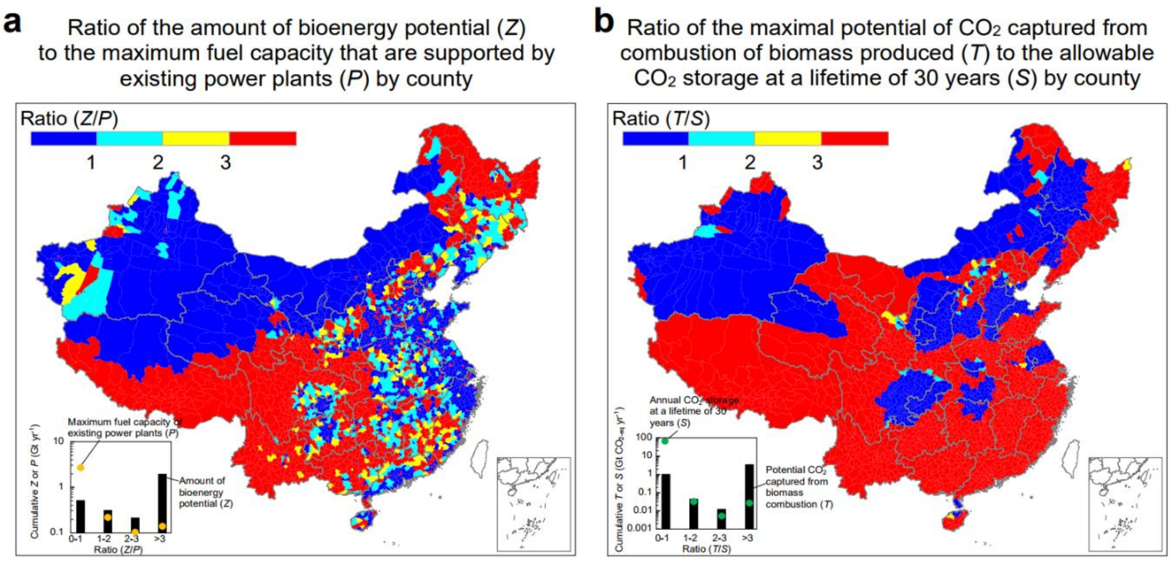

Figure S2. Spatial constraints on the capacity of power plants and carbon storage for BECCS. (**a**) Ratio of the amount of bioenergy potential (*Z*) to the maximum fuel capacity that are supported by existing power plants (*P*). A ratio of *Z/P* >1 indicates that the amount of bioenergy potential will exceed the maximum fuel capacity of existing power plants in that county. Cumulative *Z* (bars) and *P* (dots) for counties with different *Z/P* ratios are shown in the insert. (**b**) Ratio of the maximal potential of CO_2 captured from combustion of biomass produced in BECCS (*T*) to the allowable CO_2 storage at a lifetime of 30 years (*S*). A ratio of *T/S* >1 indicates that the CO_2 captured from biomass will exceed the capacity of geological carbon storage in that county. Cumulative *T* (bars) and *S* (dots) for counties with different *T/S* ratios are shown in the insert. It shows that there is only a fraction of counties with an excellent matching of biomass feedstock, electricity generation, and geological storage capacity in space.

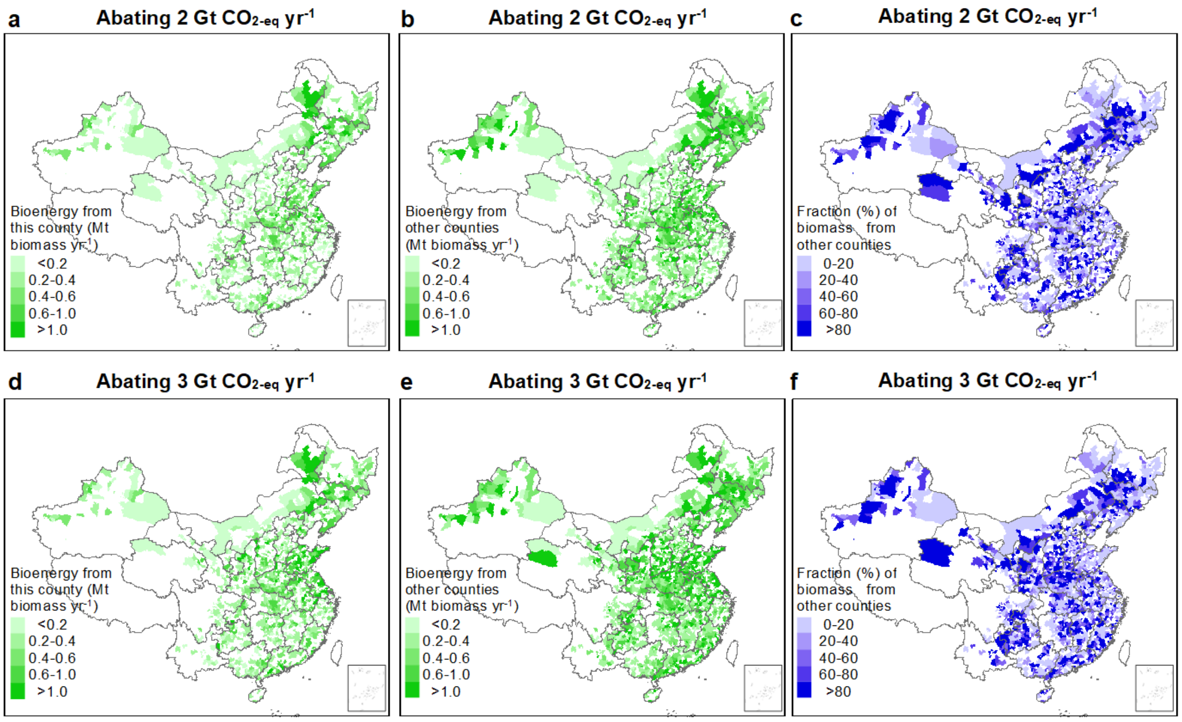

Figure S3. Sources of biomass burnt in the power plants retrofitted for BECCS in each county. (**a, d**) Amounts of biomass taken from the same county without transportation for combustion in power plants in each county. (**b, e**) Amounts of biomass transported from other counties. (**c, f**) Fraction of the burnt biomass transported from other counties or each county to abate 2 or 3 Gt $CO_{2\text{-eq}}$ yr^{-1} by BECCS in China.

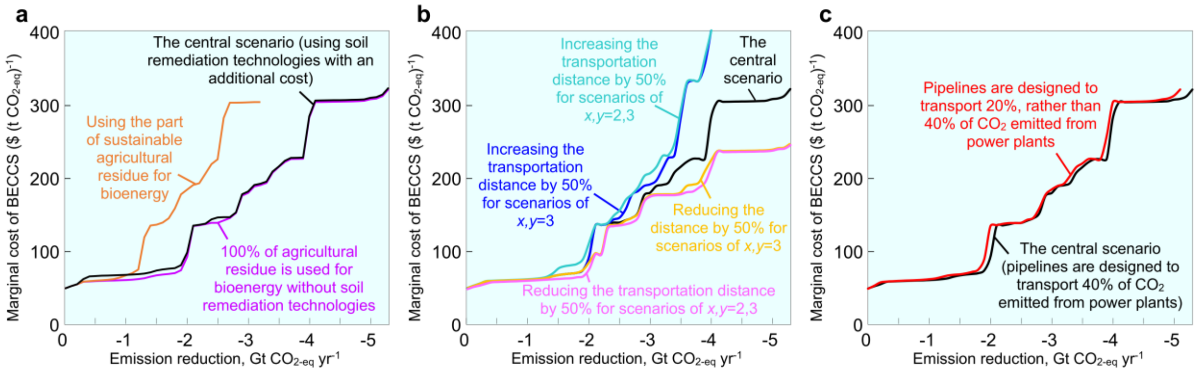

Figure S4. Sensitive tests for the marginal costs of BECCS. (**a**) Relative to the scenario *B90-2015-PC* adopting technologies at an average cost of \$ 76 ha^{-1} yr^{-1} for soil remediation (the black line), we considered a scenario using an average fraction of sustainable agricultural residues (50%) (orange), and one scenario using 100% agricultural residues without soil remediation technologies (purple). (**b**) Relative to the scenario *B90-2015-PC* with the distance of transportation of biomass and CO_2 calculated by our simple method (the black line), we designed four scenarios, where the distances of transportation for biomass and CO_2 are altered by ±50% in the scenarios of *x*,*y*=2 (the cyan and pink lines) or *x*,*y*=2,3 (the blue and orange lines). Comparing to *B90-2015-PC*, the influence on the marginal cost of BECCS is low when the abatement target is <2 Gt CO_2 yr^{-1} when long-range transportation is not used, although the influence is more significant to abate more emissions. (**c**) Relative to the scenario *B90-2015-PC*, we consider a scenario where the pipelines of CO_2 are used to transport 20% (red line), rather than 40% (blue line) of CO_2 emitted from all power plants. It reduces CO_2 flow rate in the pipelines, increases unit cost of CO_2 transport and produces a higher marginal cost of emission reduction by BECCS in our study. It suggests that the marginal cost of BECCS can be reduced (or increased) under a more (or less) ambitious target of CCS in the country.

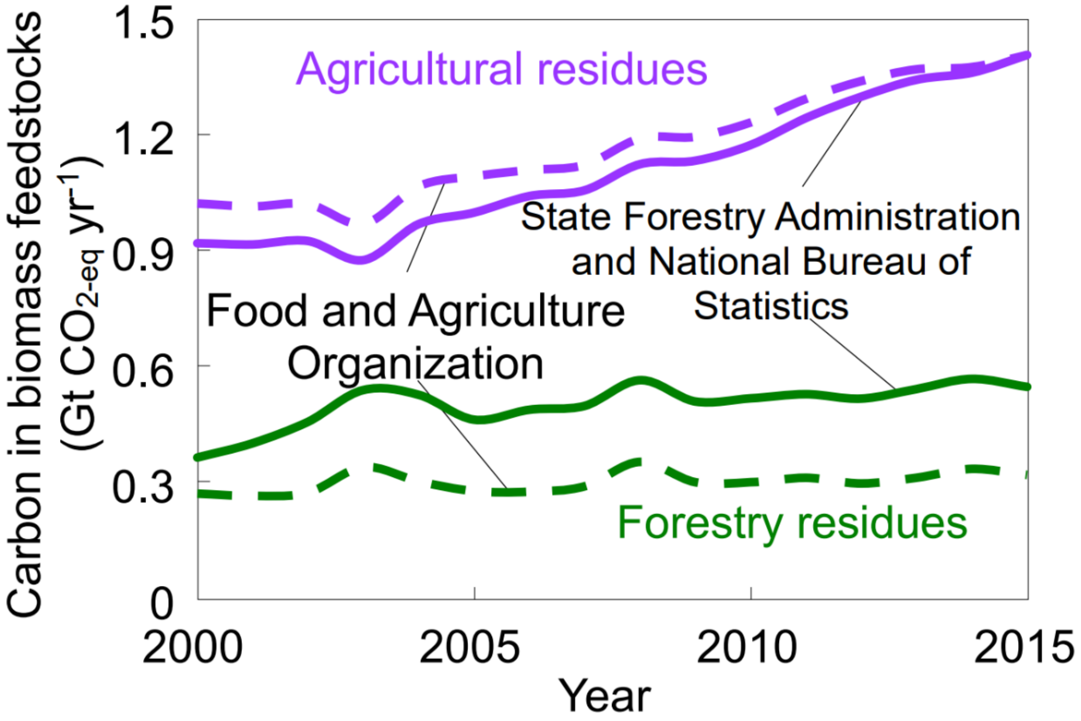

Figure S5. Biomass feedstocks from agricultural and forestry residues in China. Carbon sequestration in agricultural and forestry residues are estimated from 2000 to 2015 using data from the State Forestry Administration (SFA, 2015) of the People's Republic of China and the National Bureau of Statistics (NBSC, 2016) of the People's Republic of China, which are compared with estimation using data from the Food and Agriculture Organization of the United Nations (FAO) (FAO, 2019). Some wood products (commercial roundwood, bamboo, wood discarded by farmers and commercial fuelwood) are not included in the statistical data of FAO due to lack of data.

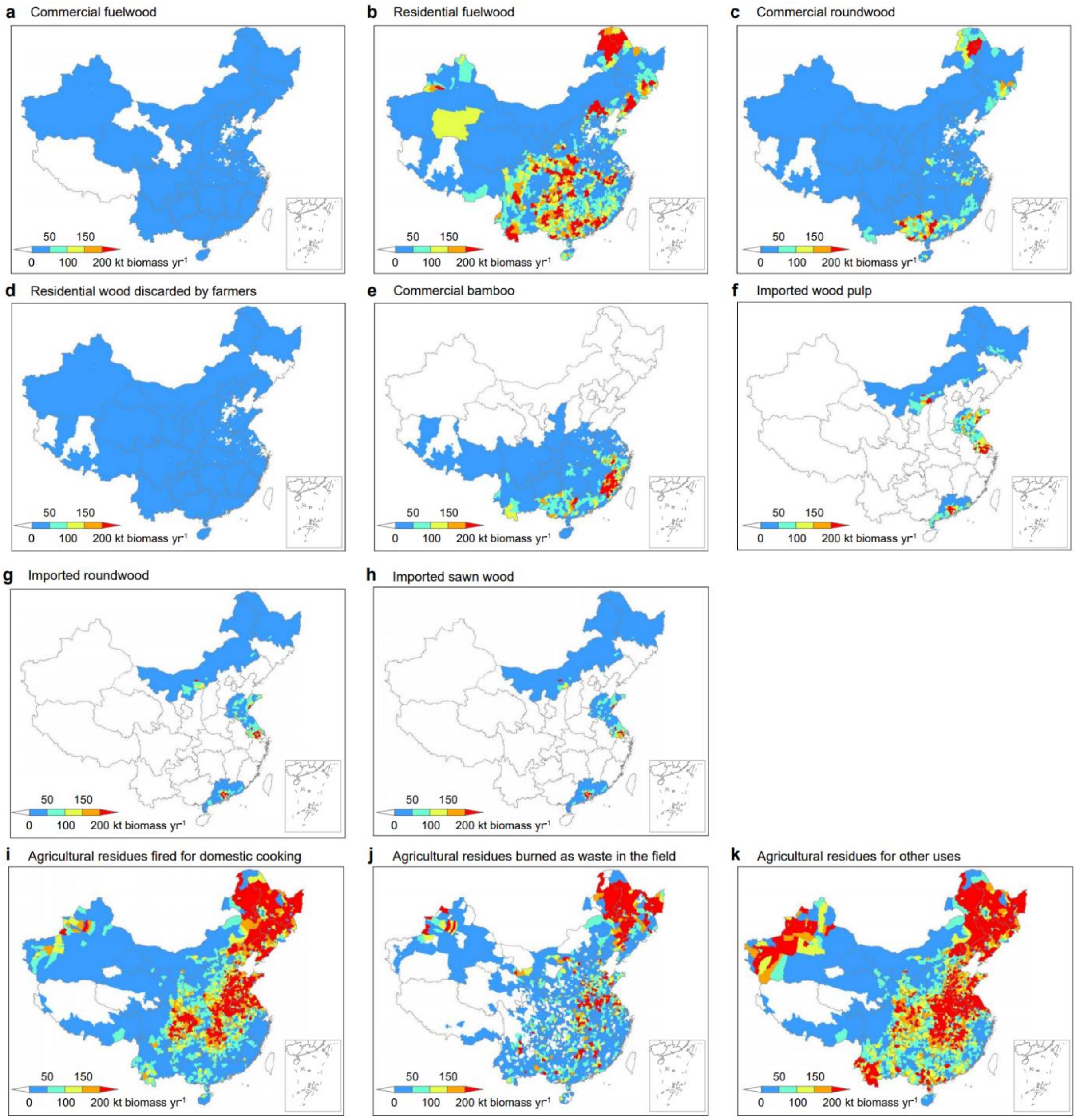

Figure S6. Spatial distributions of biomass feedstocks in China. The biomass feedstocks are shown for commercial fuelwood (**a**), residential fuelwood (**b**), commercial roundwood (**c**), residential wood discarded by farmers (**d**), commercial bamboo (**e**), imported wood pulp (**f**), imported roundwood (**g**), imported sawn wood (**h**), agricultural residues fired for domestic cooking (**i**), agricultural residues burned as waste in the field (**j**), and agricultural residues for other uses (**k**).

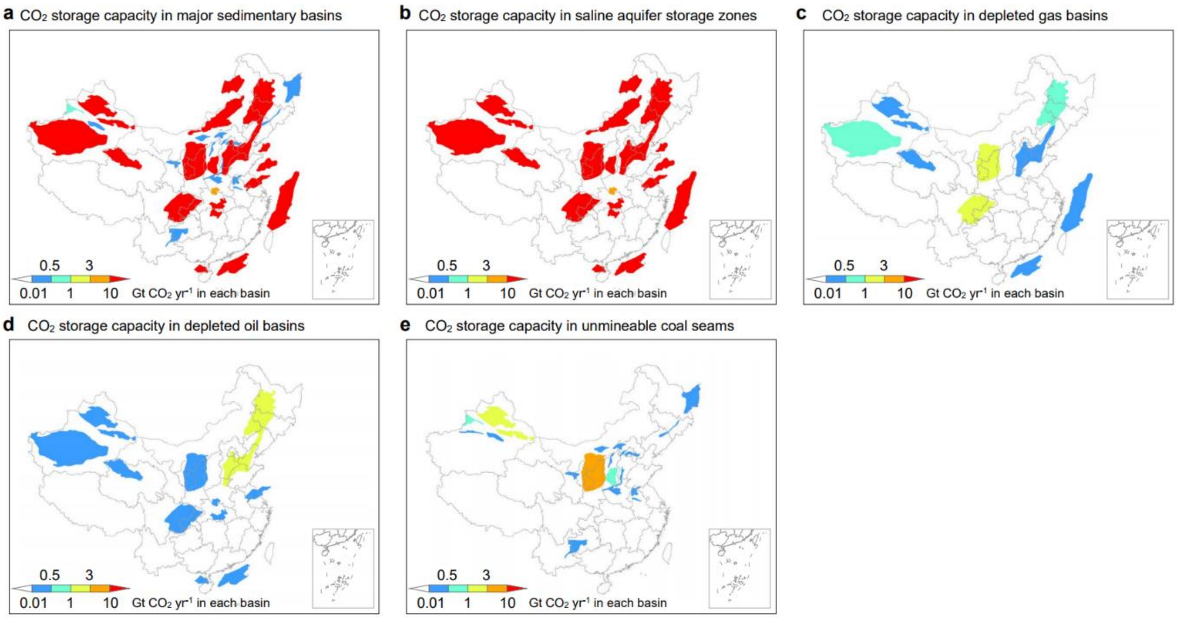

Figure S7. Spatial distributions of geological sites for carbon storage in China. Carbon storage capacities are identified for the major sedimentary basins (**a**), saline aquifer storage zones (**b**), depleted gas basins (**c**), depleted oil basins (**d**), and unmineable coal seams (**e**) in China.

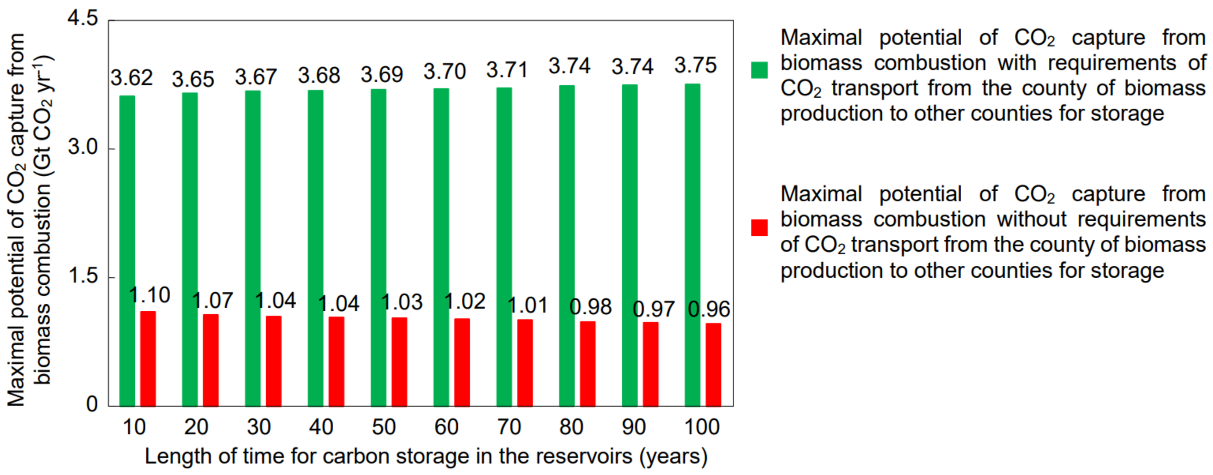

Figure S8. Impact of carbon storage length on the spatial constraints of carbon storage. The green bars show the maximal potential of CO_2 capture from biomass combustion with requirements of CO_2 transport from the county of biomass production to other counties for storage. The red bars show the maximal potential of CO_2 capture from biomass combustion without requirements of CO_2 transport from the county of biomass production to other counties for storage.

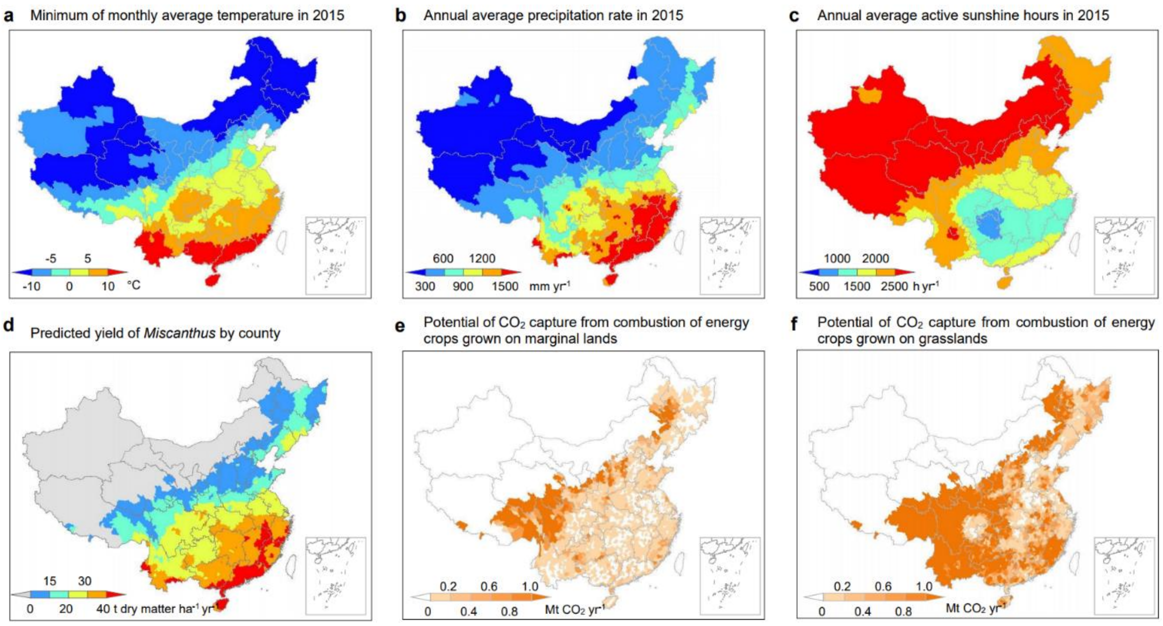

Figure S9. Spatial distribution of dedicated energy crops in China. Spatially distribution of data used to estimate the yield of *Miscanthus*, including minimum of monthly average temperature (**a**), annual average precipitation rate (**b**) and annual average active sunshine hours (**c**) in 2015. (**d**) shows the predicted yield of *Miscanthus* by county. (**e, f**) show the potential of CO_2 capture from combustion of energy crops grown on marginal lands and grasslands. In (**d**), gray area is not suitable for growing dedicated energy crops, because the minimum of monthly average temperature is below -23 °C or the annual average precipitation rate is below 400 mm yr^{-1} (Xue et al., 2016).

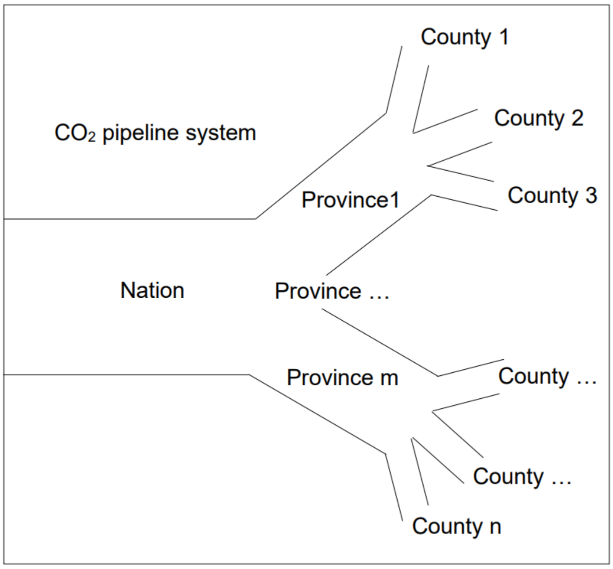

Figure S10. Framework of pipelines for CO_2 transport in China. To transport CO_2 from the site of capture to the site of storage, the pipelines in all counties in a province are converged to one provincial pipeline, and then all provincial pipelines are converged to one pipeline in the country. The unit cost of CO_2 transport is calculated as a function of the CO_2 flow rate and the length of pipeline (McCollum and Ogden, 2006). Due to increasing flow rate of CO_2, the unit cost of CO_2 transport decreases from a county pipeline to a provincial or national pipeline. In addition, Sanchez et al. (2018) suggested that the CO_2 flow rate is limited by the nominal pipeline size, and we adopted the minimal (χ^{MIN}=89 kt yr^{-1}) and maximal (χ^{MAX}= 26698 kt yr^{-1}) from that study due to lack of data in China.

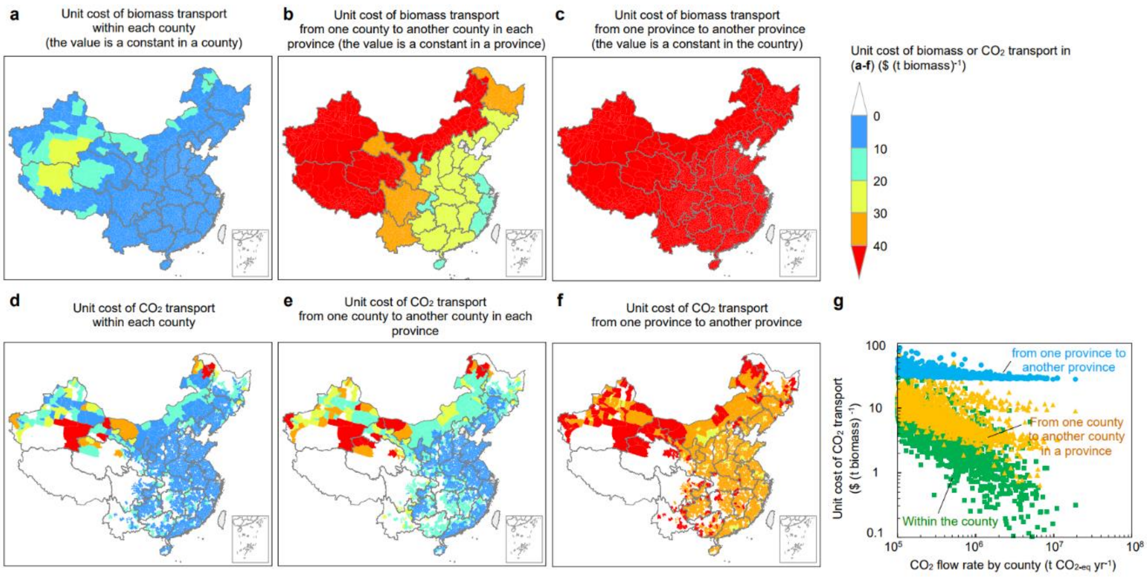

Figure S11. Unit cost of biomass and CO_2 transport in China. (**a-c**) show the unit cost of transport of biomass from the sites of collection in county *i* to a power plant in county *i* (**a**), transport of biomass from other counties in province *j* to a power plant in county *i* in province *j* (**b**), and transport of biomass from counties in other provinces to a power plant in county *i* in province *j* (**c**). (**d-f**) show the unit cost of transport of CO_2 captured in a power plant in county *i* to sites suitable for storage in county *i* (**d**), transport of CO_2 captured in a power plant in county *i* in province *j* to other counties in province *j* for storage (**e**), and transport of CO_2 captured in a power plant in county *i* to other provinces for storage (**f**). (**g**) shows the unit cost of CO_2 transport within the county (green squares), from one county to another county in a province (yellow triangles) and from one province to another province (blue circles) as a function of the CO_2 flow rate.

Table S1. Previous studies estimating the potential and marginal cost of BECCS at regional scales.

Countries	Methods	The potential of BECCS	Marginal cost	References
United States	This study combines power plant engineering, spatial optimization of biofuels, and lifecycle assessment to examine the feasibility of near-term carbon capture and sequestration (CCS) from technical and economic aspects, which suggests an opportunity of CO_2 capture at low costs ready for ethanol biorefineries in the United States	45 Mt CO_2 yr^{-1}	A carbon price of \$60 (t CO_2)$^{-1}$ abates 30 Mt CO_2 yr^{-1}; A carbon price of \$90 (t CO_2)$^{-1}$ abates 38 Mt CO_2 yr^{-1}.	Sanchez et al., 2018
Western North America	The SWITCH model is used to optimize the power sector that achieve targets of carbon mitigation.	75 Mt CO_2 yr^{-1} by substituting gasoline; 165 Mt CO_2 yr^{-1} by substituting fossil-fuel in the power sector.	\$50-1200 (t CO_2)$^{-1}$	Sanchez et al., 2015
Brazil	The potential and costs of negative emissions are assessed by developing a production system with ethanol, sugar cane, bagasse, and other agricultural residues in Brazil.	27.7 Mt CO_2 yr^{-1}	\$47.91 (t CO_2)$^{-1}$	Moreira et al., 2016
South Korea	A biophysical global forestry model (G4M) is applied to estimate the biomass feedstock, which is taken as input data to drive an engineering model (BeWhere) for optimizing the capacity and location of combined heat and power (CHP) plants. The location and capacity of forest-based bioenergy plants were combined with a map of geological suitability for carbon storage to estimate the potential of BECCS in South Korea. The costs of BECCS are not investigated.	0.13-0.24 Mt CO_2 yr^{-1}	-	Kraxner et al., 2014a
Japan	A biophysical global forestry model (G4M) is applied to estimate the biomass feedstock, which is taken as input data to drive an engineering model (BeWhere) for optimizing the capacity and location of combined heat and power (CHP) plants. The location and capacity of forest-based bioenergy plants were combined with a map of geological suitability for carbon storage to estimate the potential of BECCS in Japan. The costs of BECCS are not investigated.	1.34-1.48 Mt CO_2 yr^{-1}	-	Kraxner et al., 2014b

Table S1 (con't). Previous studies estimating the potential and marginal cost of BECCS at regional scales.

Countries	Methods	The potential of BECCS	Marginal cost	References
Europe	An energy module (TIMER) of the IMAGE Integrated Assessment modelling framework is used to explore the potential of mitigation in the industrial, transport and residential sectors, where a detailed European power model (ACE) at a high spatial and temporal resolution is used to provide a map of bioenergy supply to abate carbon emissions.	Total CO_2 emissions can be reduced by 115% in the power sector by BECCS in 2050, compared to a scenario without BECCS.	-	Deetman et al., 2013
United States	A spatially-explicit biorefinery siting and a CCS infrastructure model are combined into the Geospatial Bioenergy Systems Model (GBSM) to examine the potential of using energy crops and agricultural residues for biofuels with CCS in the United States. The CCS deployment model uses a mixed integer linear programming (MILP) optimization algorithm in the General Algebraic Modeling System (GAMS) to determines the least-cost biofuel and CCS infrastructure that meets a CO_2 capture target and/or a biofuel with CCS target. This study had optimized the transportation of CO_2 in pipelines from power plants to 439 injection sites for carbon storage, but did not account for the emissions and costs due to transportation of biomass from harvested sites to power plants.	60 Mt CO_2 yr^{-1} in 2020, 220 Mt CO_2 yr^{-1} in 2030 and 540 Mt CO_2 yr^{-1} in 2050	\$125 (t CO_2)$^{-1}$ in 2020, \$100 (t CO_2)$^{-1}$ in 2030 and \$90 (t CO_2)$^{-1}$ in 2050 for abatement of 50 Mt CO_2 yr^{-1}	Johnson et al., 2014

Table S2. Provincial bioenergy, costs and abated emissions by BECCS.

Provinces	Bioenergy (EJ yr^{-1})			Costs (billion US $ yr^{-1})			Abated emissions (Gt $CO_{2\text{-eq}}$ yr^{-1})		
	1Gt	3Gt	5Gt	1Gt	3Gt	5Gt	1Gt	3Gt	5Gt
Beijing	0.024	0.060	0.060	0.191	0.738	0.740	0.003	0.006	0.006
Chongqing	0.186	0.400	0.854	1.470	4.156	15.182	0.023	0.047	0.084
Tianjin	0.060	0.071	0.071	0.470	0.649	0.649	0.007	0.009	0.009
Anhui	0.472	1.193	1.575	3.804	12.269	18.589	0.059	0.143	0.176
Fujian	0.112	0.914	1.550	0.824	13.667	25.705	0.015	0.103	0.157
Guizhou	0.276	0.678	1.613	2.074	7.297	26.582	0.036	0.084	0.162
Hebei	0.687	1.243	1.783	5.585	12.219	21.225	0.086	0.148	0.195
Heilongjiang	0.170	1.709	2.324	1.416	21.525	36.674	0.021	0.206	0.263
Henan	0.989	1.936	2.088	8.136	18.107	20.669	0.123	0.234	0.247
Hubei	0.396	0.880	1.094	3.192	8.463	13.642	0.050	0.108	0.125
Hunan	0.227	0.765	1.198	1.785	7.718	18.437	0.029	0.095	0.134
Jiangsu	0.762	1.437	1.437	6.318	18.045	18.045	0.094	0.179	0.179
Jiangxi	0.114	0.695	0.968	0.868	7.587	13.554	0.015	0.084	0.106
Liaoning	0.356	0.769	0.932	2.834	7.289	10.001	0.045	0.094	0.108
Shaanxi	0.218	1.137	2.100	1.710	15.027	33.953	0.028	0.123	0.204
Shandong	1.119	2.096	2.227	9.248	24.612	26.822	0.139	0.254	0.265
Shanxi	0.266	1.095	1.362	2.175	14.655	19.092	0.033	0.122	0.145
Sichuan	0.273	0.952	5.407	2.285	12.553	121.746	0.035	0.115	0.494
Zhejiang	0.124	0.433	0.433	0.989	5.877	5.884	0.016	0.049	0.049
Hainan	0.034	0.182	0.182	0.251	2.456	2.457	0.004	0.019	0.019
Guangdong	0.275	1.187	1.788	2.061	17.730	28.368	0.036	0.152	0.204
Gansu	0.090	0.655	1.133	0.742	8.689	19.033	0.007	0.075	0.114
Guangxi	0.141	0.579	1.847	1.213	6.539	38.332	0.019	0.073	0.192
Jilin	0.179	1.031	1.430	1.461	10.264	20.065	0.022	0.126	0.165
Ningxia	0.064	0.201	0.201	0.528	2.554	2.562	0.008	0.023	0.023
Qinghai	0.001	0.159	3.188	0.006	2.696	76.842	0.000	0.018	0.306
Yunnan	0.085	0.574	3.492	0.736	7.792	79.377	0.011	0.070	0.313
Xinjiang	0.122	0.660	0.660	0.990	6.414	6.439	0.015	0.081	0.081
Tibet	0.000	0.013	2.741	0.000	0.217	67.064	0.000	0.002	0.239
Inner Mongolia	0.108	1.268	2.170	0.875	14.657	31.389	0.014	0.155	0.231
Shanghai	0.020	0.031	0.031	0.168	0.332	0.333	0.003	0.004	0.004
**Total**	**7.951**	**25.002**	**47.938**	**64.404**	**292.793**	**819.452**	**1**	**3**	**5**

Table S3. Acquisition and labor costs of agricultural residues, wood products and dedicated energy crops.

Abated emissions (Gt $CO_{2\text{-eq}}$ yr^{-1})	Total costs (billion US $ yr^{-1})	Acquisition costs (billion US $ yr^{-1})				Fraction of biomass acquisition costs	Labor costs (billion US $ yr^{-1})			Fraction of labor costs
		Agricultural residues	Fuelwood	Commercial wood	Energy crops		Agricultural residues	Fuelwood	Energy crops	
1	64	15	4	0	0	29%	4	2	0	8%
3	293	39	4	31	34	37%	10	2	15	9%
5	819	39	4	40	192	34%	10	2	87	12%

Table S4. Sustainable agricultural residues for bioenergy.

Methodology	Fraction for sustainable agricultural residue removal (%)	Reference
Estimated basing on US Department of Agriculture (USDA) guidelines for residue management.	40-50 (wheat, barley, rye and oats) and 40 (maize)	Kadam and McMillan, 2003
Estimated basing on the current fraction of straw usage.	30 (wheat, barley, rye, oats, maize and rapeseed)	Nikolaou et al., 2003
Estimated basing on the carbon input in the top layer of soil.	60 (wheat, barley, rye, oats and maize)	Kätterer et al., 2004
Estimated using the rain and wind erosion equations, where the input data include soil type, acres of that particular soil type, field topology characteristics (percentage low and high slopes), erodibility, and tolerable soil-loss limit.	30-70 (maize)	Graham et al., 2007
Assumed basing on empirical data.	25 (wheat, barley, rye, oats and maize)	Ericsson and Nilsson, 2006
Assumed basing on empirical data.	50 of crop residues	Fischer et al., 2010
Estimated basing on a summary of literature.	40 (wheat, rye, barely) and 50 (maize, rice and sunflower)	Scarlat et al, 2010
Estimated using the revised Universal Soil Loss Equation 2 (RUSLE2) erosion model, where the input data include management practices, soil structure, slope, slope length, temperature, precipitation, crop grain yield and supporting practices.	56-98 (corn and soybean)	Karkee et al., 2012
Estimated using the RUSLE2 model, where input data include the SSURGO soil survey database, the NRCS managed RUSLE2 climate database, the CLIGEN daily climate generator, the WINDGEN daily wind speed and direction generator, land management and crop yield.	83 (corn), 1.9 (rice) and 14 (wheat)	Muth et al., 2013
Estimated using the RUSLE2 model, where the input data include detailed site information and crop management practice.	68-76 (cotton)	Sahoo et al., 2016

Table S5. Application of soil remediation technologies in the literature. TP: Tillage practice; IRF: Increase rotational frequency; GM: Grass mulch; PM: Plastic film mulch; AC: Animal manure and compost fertilizer; IR: Irrigation; CT: Contour tillage; FA: Fallow; PA: Precision agriculture; RR: Root recycling; BC: Biochar; BI: Bioremediation.

Technologies												Reference
TP	IRF	GM	PM	AC	IR	CT	FA	PA	RR	BC	BI	
√		√	√			√						Wen et al., 2020
	√	√		√	√		√		√	√		PIRD, 2020
√												Arunrat et al., 2020
√	√	√		√					√			DAFM, 2020
√	√	√	√	√				√		√	√	Tanveer et al., 2019
√												Li et al., 2020
			√									Rahma et al., 2019
√			√									Wang et al., 2019
√												Guo et al., 2019
√				√								Li et al., 2018
√		√	√			√						Dai et al., 2018
			√									Pan et al., 2018
		√										Pan et al., 2017
			√	√								Wang et al., 2017
√	√			√	√		√	√	√	√		Chan et al., 2010

Table S6. Costs of soil remediation technologies.

Technologies	Cost ($ ha^{-1} yr^{-1})	Reference
Root mulch	58	Kuhlman et al., 2010
Grass mulch	76	Kuhlman et al., 2010
Tillage practice	78	Kuhlman et al., 2010
Contour tillage	27	Kuhlman et al., 2010
Plastic film mulch	140	Chukalla et al., 2017

Table S7. Data sources to estimate the biomass feedstocks in China.

	Biomass	Raw date sources
Forestry residues	Commercial fuelwood [a]	• Wood production from 2007 to 2015 is taken from the State Forestry Administration (SFA) of People's Republic of China (SFA, 2015), which is interpolated to years over 2000-2006 using gross domestic production (GDP) as a proxy (NBSC, 2016).
	Residential fuelwood [b]	• Wood production from 2000 to 2007 is taken from the IEA database (IEA, 2013), which is interpolated to years over 2008-2015 using rural population as a proxy (NBSC, 2016).
	Commercial roundwood [a]	• Wood production from 2000 to 2015 is taken from the SFA of the People's Republic of China (SFA, 2015).
	Residential wood discarded by farmers [a]	• Wood production from 2003 to 2015 is taken from the SFA of the People's Republic of China (SFA, 2015), which is interpolated to years over 2000-2002 using gross domestic production (GDP) as a proxy (NBSC, 2016).
	Commercial Bamboo [c]	• Bamboo production from 2000 to 2015 is taken from the SFA of the People's Republic of China (SFA, 2015).
	Imported roundwood [a]<br>Imported wood pulp [d]<br>Imported sawn wood [e]	• Wood production from 2000 to 2015 is taken from the SFA of the People's Republic of China (SFA, 2015).
Agricultural residues	Rice [f]<br>Potato [f]<br>Soybean [f]<br>Peanut [f]<br>Hemp [f]<br>Cotton [f]<br>Sugar beet [f]<br>Wheat [f]<br>Corn [f]<br>Sesame [f]<br>Rapeseed [f]	• Agricultural residues fired for domestic cooking or burnt as waste in the field are derived from the IEA database for 2000-2007 (IEA, 2013), which is interpolated to years over 2008-2015 using rural population as a proxy (NBSC, 2016).<br>• For 2000-2015, agricultural residues for other uses are derived by total production of agricultural residues (NBSC, 2016) minus the agricultural residues fired for domestic cooking and burned as waste in the field.

- For a comparison, the production of forestry residues and the consumption of agricultural residues are compiled from the FAO (FAO, 2019).
- We include the parts of forestry residues (leaves, stems, branch and bark) that can be used for combustion, thus excluding roots.
- We include the parts of agricultural residues (leaves and stems) that can be used for combustion, thus excluding grains and roots.

Table S7 (con't). Data sources to estimate the biomass feedstocks in China.

	Biomass	Raw date sources
Dedicated energy crops	Energy crops grown on marginal lands<br>Energy crops grow on grasslands	● Areas of marginal land and grasslands are identified in 2836 counties from the land cover product released by Resource and Environment Data Cloud Platform (REDCP, 2019a). To estimate the production of dedicated energy crops, the yield is predicted by a plant model based on the minimum of monthly average temperature, annual average precipitation rate and annual average active sunshine hours (Xue et al., 2016), or using a global map for the best-yield crops (Li, et al., 2020).

Spatial allocations of biomass feedstocks to 2836 counties in year 2015

- The productions of commercial fuelwood, residential fuelwood, commercial roundwood, and residential wood discarded by farmers and the production of commercial bamboo in 31 provinces are allocated to 2836 counties in China using the satellite-based forest net primary production (NPP, 1 km×1 km) (REDCP, 2019b) as a proxy.
- The amounts of imported roundwood, wood pulp and sawn wood in 5 provinces (Jiangsu, Inner-Mongolia, Heilongjiang, Shandong, and Guangdong province) are allocated to counties in China using gridded GDP (1 km×1 km) (REDCP, 2019c) as a proxy.
- The consumption of burnt agricultural residues as waste in the field are allocated to counties in China using the satellite-based gridded Moderate Resolution Imaging Spectroradiometer (MODIS) Fire Radiative Power (FRP) data (Zhou et al., 2017) as a proxy.
- The consumption of agricultural residues fired for domestic cooking and for other uses in 31 provinces is allocated to 2836 counties in China using the satellite-based crop NPP (1km×1 km) (REDCP, 2019b) as a proxy.
- For energy crops, the production of *Miscanthus* is estimated at a spatial resolution of 1km×1km based on the area of marginal lands and grasslands (REDCP, 2019a). The yield of *Miscanthus* is predicted by gridded minimum of monthly average temperature (NESSDC, 2019a), annual average precipitation rate (REDCP, 2019d) and annual average active sunshine hours (NESSDC, 2019b) using a plant model (Xue et al., 2016), which is averaged for areas by county in China.
- Biomass feedstock in eight types of forestry residues (commercial fuelwood, residential fuelwood, commercial roundwood, residential wood discarded by farmers, commercial bamboo, imported roundwood, imported wood pulp, imported sawn wood), eleven types of agricultural residues crops (rice, potato, soybean, peanut, hemp, cotton, sugar beet, wheat, corn, sesame, and rapeseed) for three types of usage (agricultural residues fired for domestic cooking, burnt as waste in the field, and used for other uses) and two types of dedicated energy crops grown on marginal lands and grasslands, GDP, electricity generation by power plants in 2015, forestry NPP, crop NPP, MODIS FRP, and capacity of carbon storage in 2836 counties are tabulated in the **Supplementary data 1**.

Notes:

[a] For commercial fuelwood, commercial roundwood, residential wood discarded by farmers, and imported roundwood, the weight of dry biomass is derived as a product of wood stem volume (m^3), a dry matter fraction of 85% (Bai, 2010), a constant stem density of 0.45 t m^{-3} (FAO, 2015), and a factor to convert stem to total weight of leaves, stems, branch and bark (1.5) (FAO, 2015; IPCC, 2006);

[b] For residential fuelwood, the weight of dry biomass is derived as a product of wood weight (t) and a dry matter fraction of 85% (Bai, 2010);

[c] For commercial bamboo, the weight of dry biomass is derived as a product of the number of bamboo, a biomass weight of 0.019 t in one bamboo (Cao, 2017), and a dry matter fraction of 85% (Bai, 2010);

[d] For imported wood pulp, the weight of dry biomass is derived as a product of wood pulp weight (t), a constant sawn wood density of 0.45 t m^{-3} (IPCC, 2006), a dry matter fraction of 85% (Bai, 2010), a factor to convert wood pulp to the weight of leaves, stems, branch and bark (5.87) (Cheng et al., 2002; FAO, 2015; IPCC, 2006);

[e] For import sawn wood, the weight of dry biomass is derived as a product of the sawn wood volume (m^3), a constant sawn wood density of 0.45 t m^{-3} (IPCC, 2006), a dry matter fraction of 85% (Bai, 2010), a factor to convert sawn wood to the weight of leaves, stems, branch and bark (2.15) (Cheng et al., 2002; FAO, 2015; IPCC, 2006);

[f] For agricultural residues, the weight of dry biomass is the product of grain production, the straw-to-grain ratio (1.05 (rice), 1.02 (potato), 1.36 (soybean), 1.46 (peanut), 1.7 (hemp), 3.66 (cotton), 0.1 (sugar beet), 1.26 (wheat), 1.63 (corn), 2.2 (sesame), and 2.03 (rapeseed)) (NDRC, 2015) and the dry matter fraction (89% (rice), 45% (potato), 91% (soybean), 94% (peanut), 83% (hemp), 83% (cotton), 45% (sugar beet), 89% (wheat), 87% (corn), 83% (sesame), and 83% (rapeseed)) (He et al., 2015; Zhou et al., 2017).

Table S8. Unit costs of BECCS in the scenario of *B90-2015-PC*.

Cost items	Unit cost (2015 US \$ (t biomass)$^{-1}$) and its uncertainty	Methods
Biomass acquisition (μ_h^a)	48.2 (±10%, agricultural residues, and fuelwood)<br>240.3 (±10%, commercial wood)<br>134.7 (±10%, energy crops)	Unit cost of biomass acquisition of agricultural residues and energy crops is calculated as a sum of costs in seeding, pesticide, machine cultivation, machine sowing, machine harvests, land occupation, labor consumption and soil remediation technologies from **Table S11** (Khanna et al., 2008; Komarek, 2013; HPBS, 2019; Kuhlman et al., 2010; Chukalla et al., 2017).<br>Because fuelwood is not commercial with a definite price in China, we assume that its unit cost is the same as agricultural residues.<br>Price of commercial wood in China (\$240.3 (t biomass)$^{-1}$) is compiled from the State Forestry Administration (SFA) of People's Republic of China (SFA, 2015).<br>Acquisition requires fixed field operations that lead to dependence of the harvest cost on the yield. Due to a lack of data, we assumed that the harvest cost for low-yielding residues (soy, potato and sugar beet) is two times of that for other residues (Komarek, 2013).
Fertilizer usage (μ_h^f)	9.1 (±10%, agricultural residues)<br>0 (±0%, wood products)<br>19.2 (±50%, energy crops)	Unit cost of biomass due to fertilizer usage is calculated as:<br>$$\mu_h^f = \sum_{m=1}^{3} C\_fer_{mh} \cdot PR\_fer_m$$<br>where *m* is the type of fertilizer (1, 2, 3 for N fertilizer (N), phosphorus pentoxide (P_2O_5) and potassium oxide (K_2O) fertilizers, respectively); *h* is the type of biomass; *C_fer* is the demand of fertilizer to sustain the element balance in soils when biomass is taken away from the land for combustion in power plants; and *PR_fer* is the price of fertilizer.<br>For *C_fer*, we adopt values of 0.0069±0.0004 t N (t crop)$^{-1}$, 0.0026±0.0005 t P_2O_5 (t crop)$^{-1}$, and 0.0128±0.0022 t K_2O (t crop)$^{-1}$ for agricultural residues (Ren et al., 2019); 0.0131±0.0090 t N (t energy crop)$^{-1}$, 0.0112±0.0072 t P_2O_5 (t energy crop)$^{-1}$, and 0.0247±0.0092 t K_2O (t energy crop)$^{-1}$ for energy crops (Lewandowski et al., 1995).<br>For *PR_fer*, we adopt values of \$584 (t N)$^{-1}$ for N, \$284 (t P_2O_5)$^{-1}$ for P_2O_5, and \$340 (t K_2O)$^{-1}$ for K_2O (Khanna et al., 2008).
Biomass transport (μ_{ix}^b)	0.2-25.1 (±20%, within a county)<br>4.7-72.0 (±53.8%, from county to county in a province)<br>173.6 (±53.8%, from province to province)	Unit cost of biomass transport is calculated as:<br>$$\mu_{ix}^b = BTD_{ix} \cdot BTP$$<br>where *i* denotes a county; *x* is the case of biomass transport (within one county, between counties in one province, and between different provinces); *BTD* is the distance of biomass transport (km), which is described in the section "Constraints on biomass supply, electricity generation and carbon storage" in Methods of the main text; *BTP* is the unit cost of biomass transport per distance and per ton biomass.<br>For *BTP*, we adopt a value of \$0.10±0.05 (t biomass)$^{-1}$ km^{-1} for China (Yu & Fan, 2009; Lu et al., 2019).<br>For *BTD*, we adopt an uncertainty of ±50% for the scenarios of *x*,*y*=2,3 in our Monte Carlo simulation.
CO_2 pipeline transport (μ_{iy}^t)	0.1-59.4 (±20%, within a county)<br>0.6-66.3 (±53.8%, from county to county in a province)<br>27.7-85.0 (±53.8%, from province to province)	Unit cost is calculated for pipelines constructed to transport CO_2 captured from power plants, using a function developed by McCollum and Ogden. (McCollum & Ogden, 2006). The detailed method is described in the section "Costs of CO_2 transport by pipeline" in Methods of the main text.<br>For the distance of transportation, we adopt an uncertainty of ±50% for the scenarios of *x*,*y*=2,3 in our Monte Carlo simulation.

Table S8 (con't). Unit costs of BECCS in the scenario of *B90-2015-PC*.

Cost items	Unit cost (2015 US $ (t biomass)$^{-1}$) and its uncertainty	Methods
Biomass pretreatment (μ_h^p)	15.3 (±20%, agricultural residues)<br>13.8 (±10%, wood products)<br>19.8 (±30%, energy crops)	Unit cost of biomass pretreatment by consuming diesel and electricity is calculated as:<br>$$\mu_h^p = DEC\_pre_h \cdot PR\_DE/LHV\_DE + ELC\_pre_h \cdot PR\_EL/1000/3.6$$<br>where *h* is the type of biomass; *DEC_pre* is the consumption of fuel energy (using diesel) in biomass pretreatment; *LHV_DE* is the lower heating value of diesel; *ELC_pre* is the consumption of electricity in biomass pretreatment; *PR_DE* is the price of diesel; and *PR_EL* is the price of electricity; and 3.6 converts 1 MWh to GJ.<br>For *DEC_pre*, we adopt values of 7.4 MJ (t biomass)$^{-1}$ for agricultural residues, 0 MJ (t biomass)$^{-1}$ for wood products, and 108 MJ (t biomass)$^{-1}$ for energy crops (Fajardy & Mac Dowell, 2017).<br>For *LHV_DE*, we adopt a value of 37.4±1.7 MJ (Litter diesel)$^{-1}$ (Fajardy & Mac Dowell, 2017).<br>For *PR_DE*, we adopt a value of $0.82±0.013 (Litter diesel)$^{-1}$ (Chang et al, 2015).<br>For *ELC_pre*, we adopt values of 911±166 MJ (t biomass)$^{-1}$ for agricultural residues, 828 MJ (t biomass)$^{-1}$ for wood products, and 1048±403 MJ (t biomass)$^{-1}$ for energy crops (Fajardy & Mac Dowell, 2017).<br>For *PR_EL*, we adopt a value of $60±10 (MWh electricity)$^{-1}$ (NEA, 2018).
Water consumption in agricultural irrigation (μ_h^g)	1.2 (±20%, agricultural residues)<br>0 (±0, wood products)<br>37.8 (±20%, energy crops)	Unit cost of water consumption in agricultural irrigation is calculated as:<br>$$\mu_h^g = WC\_A_h \cdot PR\_AW$$<br>where *h* is the type of biomass; *WC_A* is the consumption of water in irrigation; *PR_AW* is the price of agricultural water.<br>For *WC_A*, we adopt values of 4.44 t water (t biomass)$^{-1}$ for agricultural residues (HPBS, 2019), and 140 t water (t biomass)$^{-1}$ for energy crops (Fajardy & Mac Dowell, 2017).<br>For *PR_AW*, we adopt a value of $0.27±0.05 (t water)$^{-1}$ (Liu & Zou, 2014; Molinos-Senante & Donoso, 2016).
Water consumption due to co-firing and CCS in power plants (μ_h^w)	7.3 (±20%)	Unit cost of water consumption due to co-firing and CCS in power plants is calculated as:<br>$$\mu_h^w = WC\_P \cdot PR\_IW \cdot \eta \cdot \lambda_h/3.6$$<br>where *h* is the type of biomass; *WC_P* is the consumption of water in power plant; *PR_IW* is the price of industrial water (lower than the household price in China); η is the power generation efficiency in power plant; λ_h is the heat content of biomass; and 3.6 converts 1 MWh to GJ.<br>For *WC_P*, we adopt a value of 4.4 t water (MWh electricity)$^{-1}$ (Fajardy & Mac Dowell, 2017).<br>For *PR_IW*, we adopt a value of $1.25±0.25 (t water)$^{-1}$ (Liu & Zou, 2014; Molinos-Senante & Donoso, 2016).<br>For η, we adopt a value of 25.1% (Yang et al., 2019).<br>For λ_h, we adopt a value of 19±1 GJ (t biomass)$^{-1}$ (Kumar et al., 2003).

Table S8 (con't). Unit costs of BECCS in the scenario of *B90-2015-PC*.

Cost items	Unit cost (2015 US \$ (t biomass)$^{-1}$) and its uncertainty	Methods
Retrofitting power plants to be suitable for biomass co-firing and CCS in power plants (μ_h^d)	67.6 (±10%)	Unit cost of retrofitting power plants to be suitable for biomass co-firing and CCS is calculated as: $$\mu_h^d = (\frac{P \cdot CRF + FOM}{FRT \cdot CF} \cdot 1000 + VOM) \cdot \eta \cdot \lambda_h / 3.6$$ where h is the type of biomass; P is the investment cost of facilities in power plants (including the co-firing and CCS system); *CRF* is the capital recovery factor; *CF* is the capacity factor; *FOM* is annualized cost of fixed operation & maintenance; *VOM* is annualized cost of variable operation & maintenance; *FRT* is the full-load running time of power plants in one year; η is the power generation efficiency in power plants; λ_h is the heat content of biomass; and 3.6 converts 1 MWh to GJ.<br>For P, we adopt a value of \$2432 KW^{-1} (Black & Veatch, 2012; Lu et al., 2019). Unit cost of power plant retrofitting are calculated as a sum of the retrofitting cost for CCS (1457 (2015 US \$) KW^{-1}) from Lu et al. (2019) and the retrofitting cost for biomass co-firing (975.6 (2015 US \$) KW^{-1}) from Black & Veatch, (2012). Following an equation in Black & Veatch, (2012), the latter number (975.6) is calculated as 990 × 1.095× 90%, where 990 is the cost given by Black & Veatch, (2012), 1.095 is the deflation rate to convert the 2009 US dollar to the 2015 US dollar (https://wenku.baidu.com/view/f27e3eeb18e8b8f67c1cfad6195f312b3169eba8.html), and 90% is the biomass co-firing ratio.<br>For *CRF*, we adopt a value of 9% (calculated by equation 9 in the main text).<br>For *CF*, we adopt a value of 80% (Lu et al., 2019).<br>For *FOM*, we adopt a value of \$58.2 KW^{-1} yr^{-1} (Black & Veatch, 2012).<br>For *VOM*, we adopt a value of \$6.6 MWh^{-1} (Black & Veatch, 2012).<br>For *FRT*, we adopt a value of 7800 h (Koornneef et al., 2012).<br>For η, we adopt a value of 25.1% (Yang et al., 2019).<br>For λ_h, we adopt a value of 19±1 GJ (t biomass)$^{-1}$ (Kumar et al., 2003).
CO_2 capture and storage (μ_h^{cap})	82.5 (±10%, agricultural residues and energy crops)<br>82.3 (±10%, wood products)	Unit cost of CCS in power plants is calculated as: $$\mu_h^{cap} = PR\_CCS \cdot CC_h \cdot EC \cdot 3.67$$ where h is the type of biomass; *PR_CCS* is the price of CO_2 capture and storage; CC_h is carbon content in biomass; *EC* is the efficiency of CO_2 capture; 3.67 is the parameter convert C to CO_2.<br>For *PR_CCS*, we adopt a value of \$53±7 (t CO_2)$^{-1}$ (Koornneef et al., 2012; Rubin et al., 2015).<br>For CC_h, we adopt values of 47.1% for agricultural residues and energy crops (Zhang et al., 2015; Fajardy & Mac Dowell, 2017), and 47% for wood products (IPCC, 2006).<br>For *EC*, we adopt a constant value of 90% (Anderson & Peters, 2016; Lu et al., 2019).

Table S9. Unit emissions of BECCS in the scenario of *B90-2015-PC*.

Emission items	Unit emission (t CO_2 (t biomass)$^{-1}$) and its uncertainty	Methods
Biomass harvest and pretreatment (r_h^p)	0.232 (±20%, agricultural residues)<br>0.20 (±10%, wood products)<br>0.262 (±40%, energy crops)	Unit emission of biomass pretreatment is calculated as:<br>$$r_h^p = DEC\_har_h \cdot EF\_DE/Yield_h + DEC\_pre_h \cdot EF\_DE/LHV\_DE + ELC\_pre_h \cdot \varphi/1000/3.6$$<br>where h is the type of biomass; DEC_har is the consumption of fuel energy (using diesel) in biomass harvest; Yield is the yield of biomass; DEC_pre is the consumption of fuel energy (using diesel) in biomass pretreatment; LHV_DE is the lower heating value of diesel; ELC_pre is the consumption of electricity in biomass pretreatment; EF_DE is the equivalent CO_2 emission factor of diesel; φ is the equivalent CO_2 emission factor of coal in producing electricity.<br>For DEC_har, we adopt a value of 15.6 Litter diesel ha^{-1} for agricultural residues, and energy crops (Masek et al., 2015).<br>For *Yield*, we adopt values of 4.4 t biomass ha^{-1} for agricultural residues and 24.9 t biomass ha^{-1} for energy crops (NBSC, 2016, Xue et al., 2016, REDCP, 2019a).<br>For EF_DE, we adopt a value of 0.0034±0.0001 t $CO_{2\text{-eq}}$ (Litter diesel)$^{-1}$ (Fajardy & Mac Dowell, 2017).<br>For DEC_pre, we adopt values of 7.4 MJ (t biomass)$^{-1}$ for agricultural residues, 0 MJ (t biomass)$^{-1}$ for wood products, and 108 MJ (t biomass)$^{-1}$ for energy crops (Fajardy & Mac Dowell, 2017).<br>For LHV_DE, we adopt a value of 37.4±1.7 MJ (Litter diesel)$^{-1}$ (Fajardy & Mac Dowell, 2017).<br>For ELC_pre, we adopt values of 911±166 MJ (t biomass)$^{-1}$ for agricultural residues, 828 MJ (t biomass)$^{-1}$ for wood products, and 1048±403 MJ (t biomass)$^{-1}$ for energy crops (Fajardy & Mac Dowell, 2017).<br>For φ, we adopt a value of 0.85±0.10 t $CO_{2\text{-eq}}$ (MWh electricity)$^{-1}$ (Brander et al., 2011).
Production of fertilizers (r_h^{zp})	0.08 (±60%, agricultural residue)<br>0 (±0%, wood products)<br>0.18 (±60%, energy crops)	Unit emission of fertilizer production is calculated as:<br>$$r_h^{zp} = \sum_{m=1}^{3} C\_fer_{mh} \cdot EF\_fp_m$$<br>where m is the type of fertilizer (1, 2, 3 for N, P_2O_5 and K_2O fertilizers, respectively); h is the type of biomass; C_fer is the consumption of fertilizer to sustain the element balance in soils when biomass is taken away from the land for combustion in power plants; EF_fp is the equivalent CO_2 emission factor to produce one ton of fertilizer.<br>For C_fer, we adopt values of 0.0069±0.0004 t N (t crop)$^{-1}$, 0.0026±0.0005 t P_2O_5 (t crop)$^{-1}$, and 0.0128±0.0022 t K_2O (t crop)$^{-1}$ for agricultural residues (Ren et al., 2019); 0.0131±0.0090 t N (t energy crop)$^{-1}$, 0.0112±0.0072 t P_2O_5 (t energy crop)$^{-1}$ and 0.0247±0.0092 t K_2O (t energy crop)$^{-1}$ for energy crops (Lewandowski et al., 1995).<br>For EF_fp, we adopt values of 8.67±3.08 t $CO_{2\text{-eq}}$ (t N)$^{-1}$ for N, 3.21±2.26 t $CO_{2\text{-eq}}$ (t P_2O_5)$^{-1}$ for P_2O_5 and 1.16±0.43 t $CO_{2\text{-eq}}$ (t K_2O)$^{-1}$ for K_2O (Chen et al., 2015).
Application of fertilizers in agricultural lands (r_h^{za})	0.04 (±30%, agricultural residues)<br>0 (±0%, wood products)<br>0.08 (±50%, energy crops)	Unit emission of fertilizer application in agriculture is calculated as:<br>$$r_h^{za} = \sum_{m=1}^{3} C\_fer_{mh} \cdot EF\_fa_m$$<br>where m is the type of fertilizer (1, 2, 3 for N, P_2O_5 and K_2O fertilizers, respectively); h is the type of biomass; C_fer is the consumption of fertilizer to sustain the element balance in soils when biomass is taken away from the land for combustion in power plants; EF_fa is the equivalent CO_2 emission factor when one ton of fertilizer is added in soils.<br>For C_fer, we adopt values of 0.0069±0.0004 t N (t crop)$^{-1}$, 0.0026±0.0005 t P_2O_5 (t crop)$^{-1}$ and 0.0128±0.0022 t K_2O (t crop)$^{-1}$ for agricultural residues (Ren et al., 2019); 0.0131±0.0090 t N (t energy crop)$^{-1}$, 0.0112±0.0072 t P_2O_5 (t energy crop)$^{-1}$ and 0.0247±0.0092 t K_2O (t energy crop)$^{-1}$ for energy crops (Lewandowski et al., 1995).<br>For EF_fa, we adopt values of 3.60±0.80 t $CO_{2\text{-eq}}$ (t N)$^{-1}$ for N, 1.10±0.50 t $CO_{2\text{-eq}}$ (t P_2O_5)$^{-1}$ for P_2O_5 and 0.64±0.22 t $CO_{2\text{-eq}}$ (t K_2O)$^{-1}$ for K_2O (Fajardy & Mac Dowell, 2017).

Table S9 (con't). Unit emissions of BECCS in the scenario of *B90-2015-PC*.

Emission items	Unit emission (t CO_2 (t biomass)$^{-1}$) and its uncertainty	Methods
Transport of biomass from collection sites to power plants by diesel vehicles (r_{ix}^{b})	0.0001-0.02 (±5%, within a county)<br>0.004-0.06 (±50.2%, from county to county in a province)<br>0.13 (±50.2%, from province to province)	Unit emission of biomass transport from the harvest sites to the power plants by vehicles (consuming diesel) is calculated as:<br>$$r_{ix}^{b} = BTD_{ix} \cdot BEF$$<br>where *i* denotes a county; *x* is the case of biomass transport (within a county, between counties in one province, and between different provinces); *BTD* is the biomass transport distance (km), which is described in the section "Constraints on biomass supply, electricity generation and carbon storage" in Methods of the main text; *BEF* is the equivalent CO_2 emission factor to transport one ton of biomass over 1 km by vehicles (using diesel).<br>For *BEF*, we adopt a value of 0.000077±0.000003 t $CO_{2\text{-eq}}$ (t biomass)$^{-1}$ km^{-1} (Fajardy & Mac Dowell, 2017).<br>For *BTD*, we adopt an uncertainty of ±50% for the scenarios of x,y=2,3 in our Monte Carlo simulation.
Retrofitting power plants in power plants to be suitable for biomass co-firing and CCS (r_{h}^{d})	0.0004 (±10%)	Unit emission of retrofitting power plants to be suitable for biomass co-firing and CCS is calculated based on the costs to produce 1 MWh electricity and the electricity generation from bioenergy as:<br>$$r_{h}^{d}=EF\_adj \cdot \eta \cdot \lambda_h/3.6$$<br>where *h* is the type of biomass; *EF_adj* is the equivalent CO_2 emissions to produce 1 MWh electricity in the adjustment of facilities; η is the power generation efficiency in power plant; λ_h is the heat content of biomass; and 3.6 converts 1 MWh to GJ.<br>For *EF_adj*, we adopt a value of 0.00033 t $CO_{2\text{-eq}}$ (MWh electricity)$^{-1}$ (Lu et al., 2019).<br>For η, we adopt a value of 25.1% (Yang et al., 2019).<br>For λ_h, we adopt a value of 19±1 GJ (t biomass)$^{-1}$ (Kumar et al., 2003).
Land use and land cover change due to growing energy crops in the marginal lands and grasslands (r_{h}^{uc})	0 (±0%, agricultural residues and wood products)<br>0 (±10%, energy crops in marginal lands)<br>0.52 (±10%, energy crops in grasslands)	Unit emission of land use and land cover change due to growing energy crops is calculated as:<br>$$r_{h}^{uc} = LUC_h \cdot CC_h \cdot 3.67$$<br>where *h* is the type of biomass; LUC_h is the percentage of equivalent CO_2 emissions due to land use and land cover change in the transition of marginal land or grassland to land growing dedicated energy crops relative to the CO_2 sequestration in biomass; CC_h is the carbon content in biomass; 3.67 (=44/12) converts carbon to CO_2.<br>For *LUC*, we adopt values of 0 for agricultural residues and wood products; 0 for energy crops growing in marginal lands; and 30% for energy crops growing in grasslands (Fajardy & Mac Dowell, 2017).<br>For CC_h, we adopt values of 47.1% for agricultural residues and energy crops (Zhang et al., 2015; Fajardy & Mac Dowell, 2017), and 47% for wood products (IPCC, 2006).
Sequestration of CO_2 from biomass (r_{h}^{s})	1.6 (±6%)	Unit emission of CO_2 sequestration from biomass is calculated as:<br>$$r_{h}^{s}=CC_h \cdot EC \cdot 3.67$$<br>where *h* is the type of biomass; CC_h is the carbon content in biomass; *EC* is the efficiency of CO_2 capture; 3.67 (=44/12) converts carbon to CO_2.<br>For CC_h, we adopt values of 47.1% for agricultural residues and energy crops (Zhang et al., 2015; Fajardy & Mac Dowell, 2017), and 47% for wood products (IPCC, 2006).<br>For *EC*, we adopt a value of 90% (Anderson & Peters, 2016; Lu et al., 2019).

199

Table S10. Parameterization of the scenarios for BECCS in China. Scenarios include: (I) *B90-2015-PC* for retrofitting pulverized-coal (PC) plants under 90% biomass co-firing (B90) to generate electricity in 2015, (II) *B30-2015-PC* for 30% biomass co-firing (B30), (III) *B90-2015-IGCC* for transferring PC to integrated gasification combined cycle (IGCC) plants, (IV) *B30-2015-PC-EneCrop* for using dedicated energy crops (EneCrop) only, (V) *B90-2030-PC* for generating the projected electricity in 2030, (VI) *B90-2015-PC-BestCrop* for using the best-yield crops (BestCrop), (VII) *noBiomass-2015-PC* for using coal in power plants equipped with CCS, (VIII) *B90-2015-PC-noCCS* for biomass co-firing without CCS and (IX) *B90-2015-PC-routes* for considering the routes of biomass transportation between each county and the nearest ten counties.

	Scenario	Methods
Unit cost	(I)<br>*B90-2015-PC*	Our central scenario (all parameters are described in **Table S8**).
	(II)<br>*B30-2015-PC*	Different from *B90-2015-PC*, we consider 30% biomass co-firing with coal, rather than 90% co-firing. It affects the following parameters:<br>1. Unit cost of water consumption in power plants (μ_h^w) changes from \$7.3 to \$7.6 (t biomass)$^{-1}$, due to reduction in consumption of water in power plant (*WC_P*) from 4.4 to 4.2 t water (MWh electricity)$^{-1}$ (Fajardy & Mac Dowell, 2017), and power generation efficiency (η) from 25.1% to 27.3% (Yang et al., 2019);<br>2. Unit cost of retrofitting power plants (μ_h^d) decreases from \$67.6 to \$57.0 (t biomass)$^{-1}$, due to reduction in investment cost of facilities in power plants (*P*) from \$2432 to \$1782 KW^{-1} (Black & Veatch, 2012; Lu et al., 2019), annualized cost of fixed operation and maintenance for facilities (*FOM*) from \$58.2 to \$45.1 KW^{-1} yr^{-1} (Black & Veatch, 2012), and increase in power generation efficiency (η) from 25.1% to 27.3% (Yang et al., 2019).<br>All other parameters are identical to those in *B90-2015-PC*.
	(III)<br>*B90-2015-IGCC*	Different from *B90-2015-PC*, we consider transferring from PC to integrated gasification combined cycle (IGCC) system for biomass and coal co-firing in power plants. It affects the following parameters:<br>1. Unit cost of water consumption in power plants (μ_h^w) decreases from \$7.3 to \$5.7 (t biomass)$^{-1}$, due to reduction in consumption of water in power plant (*WC_P*) from 4.4 to 2.4 t water (MWh electricity)$^{-1}$ (Macknick et al., 2012), and increase in power generation efficiency (η) from 25.1% to 35.8% (Lu et al., 2019);<br>2. Unit cost of retrofitting power plants (μ_h^d) increases from \$67.6 to \$398.1 (t biomass)$^{-1}$, due to increase in investment costs of facilities in power plants (*P*) from \$2432 to \$6822 KW^{-1} (Black & Veatch, 2012), annualized cost of fixed operation and maintenance for facilities (*FOM*) from \$58.2 to \$67.1 KW^{-1} yr^{-1} (Black & Veatch, 2012), annualized cost of variable operation and maintenance for facilities (*VOM*) from 6.6 to 9.7 US \$ MWh^{-1} (Black & Veatch, 2012), capital recovery factor (*CRF*) from 0.09 to 0.174 (Lu et al., 2019), and power generation efficiency (η) from 25.1% to 35.8% (Lu et al., 2019);<br>3. Unit cost of CO_2 capture and storage (μ_h^{cap}) decreases from \$82.5 to \$63.8 (t biomass)$^{-1}$ for agricultural residues and energy crops, and from \$82.3 to \$63.6 (t biomass)$^{-1}$ for wood products, due to decrease in the price of CO_2 capture and storage (*PR_CCS*) from \$53 to \$41 (t CO_2)$^{-1}$ (Koornneef et al., 2012; Rubin et al., 2015).<br>All other parameters are identical to those in *B90-2015-PC*.
	(IV)<br>*B30-2015-PC-EneCrop*	Different from *B30-2015-PC*, we consider using dedicated energy crops (*Miscanthus*) only. The feedstocks of agricultural residues and wood products are set to be zero, and only energy crops are used for BECCS.<br>All other parameters are identical to those in *B30-2015-PC*.
	(V)<br>*B90-2030-PC*	Different from *B90-2015-PC*, we consider that the national electricity generation increases from 4.24 PWh yr^{-1} in 2015 to 9.5 PWh yr^{-1} in 2030.<br>All other parameters are identical to those in *B90-2015-PC*.
	(VI)<br>*B90-2015-PC-BestCrop*	Different from *B90-2015-PC*, for energy crops, we consider growing the best-yield crops, rather than *Miscanthus*, in China. The potential of energy crops is calculated based on a yield map of best-yield crops (Li et al., 2020).<br>All other parameters are identical to those in *B90-2015-PC*.

Table S10 (con't). Parameterization of the scenarios for BECCS in China. Scenarios include: (I) *B90-2015-PC* for retrofitting pulverized-coal (PC) plants under 90% biomass co-firing (B90) to generate electricity in 2015, (II) *B30-2015-PC* for 30% biomass co-firing (B30), (III) *B90-2015-IGCC* for transferring PC to integrated gasification combined cycle (IGCC) plants, (IV) *B30-2015-PC-EneCrop* for using dedicated energy crops (EneCrop) only, (V) *B90-2030-PC* for generating the projected electricity in 2030, (VI) *B90-2015-PC-BestCrop* for using the best-yield crops (BestCrop), (VII) *noBiomass-2015-PC* for using coal in power plants equipped with CCS, (VIII) *B90-2015-PC-noCCS* for biomass co-firing without CCS and (IX) *B90-2015-PC-routes* for considering the routes of biomass transportation between each county and the nearest ten counties.

	Scenario	Methods
Unit cost	(VII) *noBiomass-2015-PC*	Different from *B90-2015-PC*, we consider using coal only in power plants, which are retrofitted for CCS. It affects the following parameters:<br>1. Biomass feedstocks are set to be zero, and there is no cost related to biomass;<br>2. Unit cost of CO_2 pipeline cost (μ_{iy}^{t}) decreases slightly because the power generation efficiency increases from 25.1% (power generation efficiency of biomass co-firing with coal, η) to 27.9% (power generation efficiency of coal-fired plant, υ) (Yang et al., 2019) in this scenario;<br>3. Unit cost of water consumption in power plants (μ_h^{w}) increases from \$7.3 (t biomass)$^{-1}$ to 9.9 US \$ (t coal)$^{-1}$, due to changes in consumption of water (*WC_P*) from 4.4 to 4.1 t water (MWh electricity)$^{-1}$ (Fajardy & Mac Dowell, 2017), the heat content from 19 GJ (t biomass)$^{-1}$ of biomass (λ_h) to 25 GJ (t coal)$^{-1}$ of coal (λ_c) in this scenario (Cormos, 2012), and power generation efficiency from 25.1% (power generation efficiency of biomass co-firing with coal plant, η) to 27.9% (power generation efficiency of coal plant, υ) (Yang et al., 2019);<br>4. Unit cost of retrofitting power plants (μ_h^{d}) decreases from \$67.6 (t biomass)$^{-1}$ to \$65.5 (t coal)$^{-1}$, due to changes in investment costs of facilities in power plants (*P*) from \$2432 to \$1457 KW^{-1} (Lu et al., 2019), annual costs of fixed operation & maintenance (O&M) for facilities (*FOM*) from \$58.2 to \$38.5 KW^{-1} yr^{-1} (Black & Veatch, 2012), the heat content from 19 GJ (t biomass)$^{-1}$ of biomass (λ_h) to 25 GJ (t coal)$^{-1}$ of coal (λ_c) in this scenario (Cormos, 2012), and power generation efficiency from 25.1% (power generation efficiency of biomass co-firing with coal plant, η) to 27.9% (power generation efficiency of coal plant, υ) (Yang et al., 2019);<br>5. Unit cost of CO_2 capture and storage (μ_h^{cap}) decreases from \$82.5 (t biomass)$^{-1}$ for agricultural residues and energy crops and \$82.3 (t biomass)$^{-1}$ for wood products to \$78.6 (t coal)$^{-1}$, which are calculated as:<br>$$\mu_h^{cap} = PR\_CCS \cdot EC \cdot \varphi \cdot \upsilon \cdot \lambda_c / 3.6$$<br>where *PR_CCS* is the price of CO_2 capture and storage; *EC* is the efficiency of CO_2 capture; φ is the CO_2 emission factor of coal; υ is the power generation efficiency of coal-fired plant; λ_c is the heating content of coal; and 3.6 converts 1 MWh to GJ.<br>For *PR_CCS*, we adopt a value of \$53±7 ton CO_2^{-1} (Koornneef et al., 2012; Rubin et al., 2015);<br>For *EC*, we adopt a value of 90% (Anderson & Peters, 2016; Lu et al., 2019);<br>For φ, we adopt a value of 0.85±0.10 t CO_2 (MWh electricity)$^{-1}$ (Brander et al., 2011);<br>For υ, we adopt a value of 27.9% (Yang et al., 2019);<br>For λ_c, we adopt a value of 25 GJ (t coal)$^{-1}$ (Cormos, 2012).<br>All other parameters are identical to those in *B90-2015-PC*.
	(VIII) *B90-2015-PC-noCCS*	Different from *B90-2015-PC*, CCS is not equipped in power plants.<br>1. There is no cost of CCS and CO_2 transport.<br>2. Unit cost of water consumption in power plants (μ_h^{w}) changes from \$7.3 to \$8.8 (t biomass)$^{-1}$, due to changes in consumption of water (*WC_P*) from 4.4 to 3.7 t water (MWh electricity)$^{-1}$ (Fajardy & Mac Dowell, 2017), and power generation efficiency (η) from 25.1% to 36.2% (Yang et al., 2019);<br>3. Unit cost of retrofitting power plants (μ_h^{d}) decreases from \$67.6 (t biomass)$^{-1}$ to \$48.5 (t biomass)$^{-1}$, due to the reduction in investment costs of facilities in power plants (*P*) from \$2432 to \$975.4 KW^{-1} (Black & Veatch, 2012; Lu et al., 2019), annualized cost of fixed operation and maintenance (*FOM*) from \$58.2 to \$44.9 KW^{-1} yr^{-1} (Black & Veatch, 2012), annualized cost of variable operation and maintenance (*VOM*) from \$6.6 to \$4.1 MWh^{-1} (Black & Veatch, 2012), and the power generation efficiency (η) from 25.1% to 36.2 % (Yang et al., 2019).<br>All other parameters are identical to those in *B90-2015-PC*.

Table S10 (con't). Parameterization of the scenarios for BECCS in China. Scenarios include: (I) *B90-2015-PC* for retrofitting pulverized-coal (PC) plants under 90% biomass co-firing (B90) to generate electricity in 2015, (II) *B30-2015-PC* for 30% biomass co-firing (B30), (III) *B90-2015-IGCC* for transferring PC to integrated gasification combined cycle (IGCC) plants, (IV) *B30-2015-PC-EneCrop* for using dedicated energy crops (EneCrop) only, (V) *B90-2030-PC* for generating the projected electricity in 2030, (VI) *B90-2015-PC-BestCrop* for using the best-yield crops (BestCrop), (VII) *noBiomass-2015-PC* for using coal in power plants equipped with CCS, (VIII) *B90-2015-PC-noCCS* for biomass co-firing without CCS and (IX) *B90-2015-PC-routes* for considering the routes of biomass transportation between each county and the nearest ten counties.

	Scenario	Methods
Unit cost	(IX) *B90-2015-PC-routes*	Different from *B90-2015-PC*, this scenario considers the routes of biomass transportation between each county and the nearest ten counties or other counties in this province or other provinces.<br>Unit cost of transport of biomass from collection sites to power plants by diesel vehicles (μ_{ix}^{b}) depends on the distance of transportation. For the nearest ten counties, the distance of transportation is calculated based on the latitude and longitude of the center of the two counties. For other counties, we use the average distance between county and other counties.<br>r_{ix}^{b} is \$0.1-\$25.1, \$0.1-\$85.4, \$0.3-\$86.1, \$0.6-\$86.9, \$0.7-\$87.4, \$0.7-\$89.2, \$0.8-\$90.4, \$1.1-\$90.4, \$1.3-\$91.6, and \$1.9-\$92.3 (t biomass)$^{-1}$ for biomass transported from the nearest ten counties, and \$2.2-\$165.6 (t biomass)$^{-1}$ for biomass transported from other counties in this province, and \$110.9-\$435.6 (t biomass)$^{-1}$ for biomass transported from other counties in other provinces.
Unit emission	(I) *B90-2015-PC*	Our central scenario (all parameters are described in **Table S9**).
	(II) *B30-2015-PC*	Different from *B90-2015-PC*, we consider 30% biomass co-firing with coal, rather than 90% co-firing. Unit emission of retrofitting power plants (r_{h}^{d}) increase from 0.0004 to 0.0005 t CO_2 (t biomass)$^{-1}$, due to an increase in power generation efficiency (η) from 25.1% to 27.3% (Yang et al., 2019).<br>All other parameters are identical to those in *B90-2015-PC*.
	(III) *B90-2015-IGCC*	Different from *B90-2015-PC*, we consider integrated gasification combined cycle (IGCC) plants, rather than pulverized-coal (PC) plants. Unit emission of retrofitting power plants (r_{h}^{d}) changed from 0.0004 to 0.0017 t CO_2 (t biomass)$^{-1}$, due to the change in equivalent CO_2 emissions to produce 1 MWh electricity in the adjustment of facilities (*EF_adj*) from 0.00033 to 0.00088 t CO_{2eq} (MWh electricity)$^{-1}$ (Lu et al., 2019), and electricity efficient (η) from 25.1% to 35.8% (Lu et al., 2019) in this scenario.<br>All other parameters are identical to those in *B90-2015-PC*.
	(IV) *B30-2015-PC-EneCrop*	Different from *B30-2015-PC*, we consider using dedicated energy crops (*Miscanthus*) only. The feedstocks of agricultural residues and wood products are set to be zero, and only energy crops are used for BECCS.<br>All other parameters are identical to those in *B30-2015-PC*.
	(V) *B90-2030-PC*	Different from *B90-2015-PC*, we consider that the national electricity generation increases from 4.24 PWh yr^{-1} in 2015 to 9.5 PWh yr^{-1} in 2030.<br>All other parameters are identical to those in *B90-2015-PC*.
	(VI) *B90-2015-PC-BestCrop*	Different from *B90-2015-PC*, for energy crops, we consider growing the best-yield crops, rather than *Miscanthus*, in China. The potential of energy crops is calculated based on a yield map of best-yield crops (Li et al., 2020).<br>All other parameters are identical to those in *B90-2015-PC*.

Table S10 (con't). Parameterization of the scenarios for BECCS in China. Scenarios include: (I) *B90-2015-PC* for retrofitting pulverized-coal (PC) plants under 90% biomass co-firing (B90) to generate electricity in 2015, (II) *B30-2015-PC* for 30% biomass co-firing (B30), (III) *B90-2015-IGCC* for transferring PC to integrated gasification combined cycle (IGCC) plants, (IV) *B30-2015-PC-EneCrop* for using dedicated energy crops (EneCrop) only, (V) *B90-2030-PC* for generating the projected electricity in 2030, (VI) *B90-2015-PC-BestCrop* for using the best-yield crops (BestCrop), (VII) *noBiomass-2015-PC* for using coal in power plants equipped with CCS, (VIII) *B90-2015-PC-noCCS* for biomass co-firing without CCS and (IX) *B90-2015-PC-routes* for considering the routes of biomass transportation between each county and the nearest ten counties.

	Scenario	Methods
Unit emission	(VII)<br>*noBiomass-2015-PC*	Different from *B90-2015-PC*, we consider using coal only in power plants, which are retrofitted for CCS. It affects the following parameters:<br>1. Biomass feedstocks are set to be zero;<br>2. Unit emission of retrofitting power plants (r_h^d) increases from 0.0004 t CO_2 (t biomass)$^{-1}$ to 0.0006 t CO_2 (t coal)$^{-1}$, due to increases in power generation efficiency from 25.1% (power generation efficiency of biomass co-firing with coal plant, η) to 27.9% (power generation efficiency of coal plant, υ) (Yang et al., 2019), and the heat content to 25 GJ (t coal)$^{-1}$ of coal (λ_c) in this scenario (Cormos, 2012).<br>3. Unit emission of captured CO_2 from coal power plant is 1.5 t CO_2 (t coal)$^{-1}$ (Brander et al., 2011; Cormos, 2012; Yang et al., 2019; Anderson & Peters, 2016; Lu et al., 2019);<br>All other parameters are identical to those in *B90-2015-PC*.
	(VIII)<br>*B90-2015-PC-noCCS*	Different from *B90-2015-PC*, CCS is not equipped in power plants.<br>There is no emission related to CCS.<br>All other parameters are identical to those in *B90-2015-PC*.
	(IX)<br>*B90-2015-PC-routes*	Different from *B90-2015-PC*, this scenario considers the routes of biomass transportation between each county and the nearest ten counties or other counties in this province or other provinces.<br>Unit emission of transport of biomass from collection sites to power plants by diesel vehicles (r_{ix}^b) depends on the distance of transportation. For the nearest ten counties, the distance of transportation is calculated based on the latitude and longitude of the center of the two counties. For other counties, we use the average distance between county and other counties.<br>r_{ix}^b is 0.0001-0.019, 0.0001-0.066, 0.00021-0.066, 0.00045-0.067, 0.00050-0.067, 0.00052-0.069, 0.00060-0.070, 0.00088-0.070, 0.0010-0.071, and 0.0014-0.071 t CO_2 (t biomass)$^{-1}$ for biomass transported from the nearest ten counties, and 0.0017-0.13 t CO_2 (t biomass)$^{-1}$ for biomass transported from other counties in this province, and 0.085-0.34 t CO_2 (t biomass)$^{-1}$ for biomass transported from other counties in other provinces.

Table S11. Costs of growing agricultural crops and dedicated energy crops.

Costs	Agricultural crops (US \$ (t biomass)$^{-1}$)	Dedicated energy crops (US \$ (t biomass)$^{-1}$)
Seeding	5.1 (Komarek, 2013; HPBS, 2019)	1.6 (Khanna et al., 2008)
Pesticide	2.4 (Komarek, 2013; HPBS, 2019)	0.4 (Khanna et al., 2008)
Machine cultivation	5.2 (Komarek, 2013; HPBS, 2019)	0.2 (Khanna et al., 2008)
Machine sowing	1.6 (Komarek, 2013; HPBS, 2019)	1.6 (Khanna et al., 2008)
Machine harvest	4.3 (Komarek, 2013; HPBS, 2019)	48.9 (Khanna et al., 2008)
Land cost	-	18.1 (Khanna et al., 2008)
Labor cost	12.4 (Komarek, 2013; HPBS, 2019)	60.9 (Khanna et al., 2008)
Soil remediation technologies	17.2 (Kuhlman et al., 2010; Chukalla et al., 2017)	3.1 (Kuhlman et al., 2010; Chukalla et al., 2017)
Total cost	48.2	134.7

Supplementary References:

1. Anderson, K. & Peters, G. The trouble with negative emissions. *Science* **354**, 182-183 (2016).
2. Arunrat, N., Pumijumnong, N., Sereenonchai, S. & Chareonwong, U. Factors Controlling Soil Organic Carbon Sequestration of Highland Agricultural Areas in the Mae Chaem Basin, Northern Thailand. *Agronomy*, **10**, 305 (2020).
3. Bai, Y. F. Carbon Stocks of Usable Wood Products in China. PhD thesis, Chinese Academy of Forestry Sciences (2010); http://cdmd.cnki.com.cn/Article/CDMD-82201-2010264555.htm.
4. Black & Veatch. *Cost and Performance Data for Power Generation Technologies.* https://refman.energytransitionmodel.com/publications/1921 (2012).
5. Brander, M., Sood, A., Wylie, C., Haughton, A. & Lovell, J. *Technical Paper| Electricity-specific Emission Factors for Grid Electricity*. https://ecometrica.com/assets/Electricity-specific-emission-factors-for-grid-electricity.pdf (2011).
6. Cao, F. M. Biomass and Carbon Storage of Bamboo Forest Ecosystem in Taojiang. PhD thesis, Central South University of Forestry and Technology (2017); http://cdmd.cnki.com.cn/Article/CDMD-10538-1017118194.htm.
7. Chan, K. Y., Oates, A., Liu, D. L., Li, G. D. & Conyers, M. K. *A Farmer's Guide To Increasing Soil Organic Carbon Under Pastures.* https://www.dpi.nsw.gov.au/_data/assets/pdf_file/0014/321422/A-farmers-guide-to-increasing-Soil-Organic-Carbon-under-pastures.pdf (2010).
8. Chang, Y., Huang, R., Ries, R. J., & Masanet, E. Life-cycle comparison of greenhouse gas emissions and water consumption for coal and shale gas fired power generation in China. *Energy* **86**, 335-343 (2015).
9. Chen, S., Lu, F. & Wang, X. Estimation of greenhouse gases emission factors for China's nitrogen, phosphate, and potash fertilizers. *Acta Ecologica Sinica* **35**, 6371-6383 (2015).
10. Cheng, B., Song, W. & Tian, M. Analysis of China's imports and exports of major timber products in 2002, *Journal of Beijing Forestry University (Social Sciences)* **2**, 38-41 (2002).
11. Chukalla, A. D., Krol, M. S. & Hoekstra, A. Y. Marginal cost curves for water footprint reduction in irrigated agriculture: guiding a cost-effective reduction of crop water consumption to a permit or benchmark level. *Hydrol. Earth Syst. Sci.* **21**, 3507 (2017).
12. Cormos, C. C. Integrated assessment of IGCC power generation technology with carbon capture and storage (CCS). *Energy* **42**, 434-445 (2012).
13. Dai, C. et al. Exploring optimal measures to reduce soil erosion and nutrient losses in southern China. *Agric. Water Manage.* **210**, 41-48 (2018).
14. Deetman, S. et al. Deep greenhouse gas emission reductions in Europe: Exploring different options. *Energy Policy* **55**, 152-164 (2013).
15. Department of Agriculture, Food, and the Marine (DAFM). Maintenance of Soil Organic Matter. https://www.agriculture.gov.ie/farmerschemespayments/crosscompliance/soilorganicmatter/ (2020).
16. Ericsson, K. & Nilsson, L. J. Assessment of the potential biomass supply in Europe using a resource-focused approach. *Biomass Bioenergy* **30**, 1-15 (2006).
17. Fajardy, M. & Mac Dowell, N. Can BECCS deliver sustainable and resource efficient negative emissions? *Energy Environ. Sci.* **10**, 1389-1426 (2017).
18. Fischer, G. et al. Biofuel production potentials in Europe: Sustainable use of cultivated land and pastures, Part II: Land use scenarios. *Biomass Bioenergy* **34**, 173-187 (2010).
19. Food and Agriculture Organization (FAO) of the United Nations. *Forestry Production and Trade and Crops.* http://www.fao.org/faostat/en/#data (2019).
20. Food and Agriculture Organization (FAO) of the United Nations. *Global Forest Resources Assessment 2015*. http://www.fao.org/forest-resources-assessment/current-assessment/country-reports/zh/ (2015).
21. Graham, R. L., Nelson, R., Sheehan, J., Perlack, R. D. & Wright, L. L. Current and potential US corn

stover supplies. *Agron. J.* **99**, 1-11 (2007).

22. Guo, S. et al. Cross-ridge tillage decreases nitrogen and phosphorus losses from sloping farmlands in southern hilly regions of China. *Soil Tillage Res.* **191**, 48-56 (2019).

23. He, M., Wang, X., Han, L., Feng, X. & Mao, X. Emission inventory of crop residues field burning and its temporal and spatial distribution in Sichuan province. *Environ. Sci.* **36**, 1208-1216 (2015).

24. Henan Province Bureau of Statistics (HPBS). *Investigation and Analysis of Wheat Cost and Benefit in Henan Province in 2019*. http://www.ha.stats.gov.cn/2019/07-29/1371397.html (2019).

25. Intergovernmental Panel on Climate Change (IPCC). *2006 Guidelines for National Greenhouse Gas Inventories* (eds Eggleston, H. S., Buendia, L., Miwa, K., Ngara, T. & Tanabe, K.) Vol. 2 (Institute for Global Environmental Strategies Japan, 2006).

26. International Energy Agency (IEA). *World energy statistics and balances*. http://www.oecd-ilibrary.org/statistics (2013).

27. Johnson, Nils, Nathan Parker, and Joan Ogden. How negative can biofuels with CCS take us and at what cost? Refining the economic potential of biofuel production with CCS using spatially-explicit modeling. *Energy Procedia* **63**, 6770-6791 (2014).

28. Kadam, K. L. & McMillan, J. D. Availability of corn stover as a sustainable feedstock for bioethanol production. *Bioresour. Technol.* **88**, 17-25 (2003).

29. Karkee, M., McNaull, R. P., Birrell, S. J. & Steward, B. L. Estimation of optimal biomass removal rate based on tolerable soil erosion for single-pass crop grain and biomass harvesting system. *Trans. ASABE* **55**, 107-115 (2012).

30. Kätterer, T., Andrén, O. & Persson, J. The impact of altered management on long-term agricultural soil carbon stocks–a Swedish case study. *Nutr. Cycling Agroecosyst.* **70**, 179-188 (2004).

31. Khanna, M., Dhungana, B. & Clifton-Brown, J. Costs of producing *Miscanthus* and *Switchgrass* for bioenergy in Illinois. *Biomass Bioenergy* **32**, 482-493 (2008).

32. Komarek, A. M. Costs and benefits of crop residue retention in a Chinese subsistence farming system. No. 424-2016-27114, (2013).

33. Koornneef, J. et al. Global potential for biomass and carbon dioxide capture, transport and storage up to 2050. *Int. J. Greenhouse Gas Control* **11**, 117-132 (2012).

34. Kraxner, F. et al. BECCS in South Korea—Analyzing the negative emissions potential of bioenergy as a mitigation tool. *Renewable Energy* **61**, 102-108 (2014a).

35. Kraxner, F. et al. Energy resilient solutions for Japan-a BECCS case study. *Energy Procedia* **61**, 2791-2796 (2014b).

36. Kuhlman, T., Reinhard, S. & Gaaff, A. Estimating the costs and benefits of soil conservation in Europe. *Land Use Policy* **27**, 22-32 (2010).

37. Kumar, A., Cameron, J. B. & Flynn, P. C. Biomass power cost and optimum plant size in western Canada. *Biomass Bioenergy* **24**, 445-464 (2003).

38. Lewandowski, I., Kicherer, A. & Vonier, P. CO_2-balance for the cultivation and combustion of *Miscanthus*. *Biomass Bioenergy* **8**, 81-90 (1995).

39. Li, M. et al. Cropland physical disturbance intensity: plot-scale measurement and its application for soil erosion reduction in mountainous areas. *Journal of Mountain Science* **15**, 198-210 (2018).

40. Li, T. et al. Exploring the interaction of surface roughness and slope gradient in controlling rates of soil loss from sloping farmland on the Loess Plateau of China. *Hydrol. Processes* **34**, 339-354 (2020).

41. Li, W. et al. Mapping the yields of lignocellulosic bioenergy crops from observations at the global scale. *Earth Syst. Sci. Data Discuss.* **12**, 789-804 (2020).

42. Liu, X. & Zou, C. Calculating and forecasting shadow prices of all kinds of water in China and its nine major river basins. *Advances in Science and Technology of Water Resources* **34**, 10-15 (2014).

43. Lu, X. et al. Gasification of coal and biomass as a net carbon-negative power source for environment-friendly electricity generation in China. *Proc. Natl. Acad. Sci. U.S.A.* **116**, 8206-8213 (2019).

44. Macknick, J., Newmark, R., Heath, G. & Hallett, K. C. Operational water consumption and withdrawal factors for electricity generating technologies: a review of existing literature. *Environ. Res. Lett.*, **7**, 045802. (2012).

45. Masek, J., Novak, P., & Pavlicek, T. Evaluation of combine harvester fuel consumption and operation costs. *In 14th International Scientific Conference Engineering for Rural Development Proceedings* **14**, 78-83 (2015).

46. McCollum, D. L. & Ogden, J. M. Techno-Economic Models for Carbon Dioxide Compression, Transport, and Storage & Correlations for Estimating Carbon Dioxide Density and Viscosity. Institute of Transportation Studies University of California-Davis. UCD-ITS-RR-06-14 (2006).

47. Molinos-Senante, M. & Donoso, G. Water scarcity and affordability in urban water pricing: A case study of Chile. *Utilities Policy* **43**, 107-116 (2016).

48. Moreira, J. R., Romeiro, V., Fuss, S., Kraxner, F., & Pacca, S. A. BECCS potential in Brazil: Achieving negative emissions in ethanol and electricity production based on sugar cane bagasse and other residues. *Appl. Energy* **179**, 55-63 (2016).

49. Muth Jr, D. J., Bryden, K. M. & Nelson, R. G. Sustainable agricultural residue removal for bioenergy: A spatially comprehensive US national assessment. *Appl. Energy* **102**, 403-417 (2013).

50. National Bureau of Statistics of the People's Republic of China (NBSC). *China Statistics Yearbook 2001-2016* (China Statistics Press, Beijing, 2016).

51. National Development and Reform Commission (NDRC). *National Utilization and Burning of Straw in 2012*. https://www.ndrc.gov.cn/fggz/hjyzy/zyzhly/201512/t20151216_1135517.html (2015).

52. National Earth System Science Data Center (NESSDC). *Monthly Mean Temperature Dataset of 1km resolution in China in 2015*. http://www.geodata.cn/data/datadetails.html?dataguid=22363132729806&docId=3670 (2019a).

53. National Earth System Science Data Center (NESSDC). *Monthly Sunshine Hours Dataset of 1km resolution in China in 2015*. http://www.geodata.cn/data/datadetails.html?dataguid=278608814236557&docId=2672 (2019b).

54. National Energy Administration (NEA) of the People's Republic of China. *Regulatory Bulletin on the State Power Price in 2017*. http://www.nea.gov.cn/137519800_15391333051221n.pdf (2018).

55. Nikolaou, A., Remrova, M. & Jeliazkov, I. Lot 5: Bioenergy's Role in the EU Energy Market. *Biomass Availability in Europe* (2003).

56. Pan, D. et al. Application rate influences the soil and water conservation effectiveness of mulching with chipped branches. *Soil Sci. Soc. Am. J.* **82**, 447-454 (2018).

57. Pan, D. et al. Effect of plant cover type on soil water budget and tree photosynthesis in jujube orchards. *Agric. Water Manage.* **184**, 135-144 (2017).

58. Primary Industries and Regional Development (PIRD). *Managing Soil Organic Carbon On Western Australian Farms.* https://www.agric.wa.gov.au/soil-carbon/managing-soil-organic-carbon-western-australian-farms (2020)

59. Rahma, A. E., Warrington, D. N. & Lei, T. Efficiency of wheat straw mulching in reducing soil and water losses from three typical soils of the Loess Plateau, China. *International Soil and Water Conservation Research* **7**, 335-345 (2019).

60. Ren, J., Yu, P. & Xu, X. Straw utilization in China—status and recommendations. *Sustainability* **11**, 1-17 (2019).

61. Resource and Environment Data Cloud Platform (REDCP). *Annual Precipitation Spatial Interpolation data set in China in 2015*. http://www.resdc.cn/data.aspx?DATAID=229 (2019d).

62. Resource and Environment Data Cloud Platform (REDCP). *Gross Domestic Product Data in China in 2015*. http://www.resdc.cn/data.aspx?DATAID=252 (2019c).

63. Resource and Environment Data Cloud Platform (REDCP). *Net Primary Production Data in China in 2015*. http://www.resdc.cn/data.aspx?DATAID=204 (2019b).

64. Resource and Environment Data Cloud Platform (REDCP). *Remote Sensing Monitoring Data of Land-use in China in 2015*. http://www.resdc.cn/data.aspx?DATAID=184 (2019a).

65. Rubin, E. S., Davison, J. E. & Herzog, H. J. The cost of CO_2 capture and storage. *Int. J. Greenhouse Gas Control* **40**, 378-400 (2015).

66. Sahoo, K., Hawkins, G. L., Yao, X. A., Samples, K. & Mani, S. GIS-based biomass assessment and supply logistics system for a sustainable biorefinery: A case study with cotton stalks in the Southeastern US. *Appl. Energy* **182**, 260-273 (2016).

67. Sanchez, D. L., Johnson, N., McCoy, S. T., Turner, P. A. & Mach, K. J. Near-term deployment of carbon capture and sequestration from biorefineries in the United States. *Proc. Natl. Acad. Sci. U.S.A.* **115**, 4875-4880 (2018).

68. Sanchez, D. L., Nelson, J. H., Johnston, J., Mileva, A. & Kammen, D. M. Biomass enables the transition to a carbon-negative power system across western North America. *Nat. Clim. Chang.* **5**, 230–234 (2015).

69. Scarlat, N., Martinov, M. & Dallemand, J. F. Assessment of the availability of agricultural crop residues in the European Union: potential and limitations for bioenergy use. *Waste Manage.* **30**, 1889-1897 (2010).

70. Tanveer, S. K., Lu, X., Hussain, I. & Sohail, M. Soil Carbon Sequestration through Agronomic Management Practices. *In CO_2 Sequestration*. IntechOpen (2019).

71. The State Forestry Administration (SFA) of the People's Republic of China. *China Forestry Statistical Yearbook 2000-2015* (China Forestry Press, Beijing, 2015).

72. Wang, L. et al. Soil C and N dynamics and hydrological processes in a maize-wheat rotation field subjected to different tillage and straw management practices. *Agric. Ecosyst. Environ.* **285**, 106616 (2019).

73. Wang, Y. et al. Modelling soil detachment of different management practices in the red soil region of China. *Land Degrad. Dev.* **28**, 1496-1505 (2017).

74. Wen, X. & Zhen, L. Soil erosion control practices in the Chinese Loess Plateau: A systematic review. *Environ. Dev.* **34**, 100493 (2020).

75. Xue, S., Lewandowski, I., Wang, X. & Yi, Z. Assessment of the production potentials of *Miscanthus* on marginal land in China. *Renewable Sustainable Energy Rev.* **54**, 932-943 (2016).

76. Yang, B., Wei, Y. M., Hou, Y., Li, H. & Wang, P. Life cycle environmental impact assessment of fuel mix-based biomass co-firing plants with CO_2 capture and storage. *Appl. Energy* **252**, 113483. (2019).

77. Yu, X. & Fan, F. Simulation analysis on fuel collection, processing, storage and transportation used in straw power plant in China, *Transactions of the Chinese Society of Agricultural Engineering* **25**, 215-219 (2009).

78. Zhang, W. et al. Relative contribution of maize and external manure amendment to soil carbon sequestration in a long-term intensive maize cropping system. *Sci. Rep.* **5**, 10791 (2015).

79. Zhou, Y. et al. A comprehensive biomass burning emission inventory with high spatial and temporal resolution in China. *Atmos. Chem. Phys.* **17**, 2839-2864 (2017).
