# Supplementary material for: Spatially explicit analysis identifies significant potential for bioenergy with carbon capture and storage in China
Source: Nat Commun. 2021 May 26;12:3159. doi: 10.1038/s41467-021-23282-x (PMC8154910; doi:10.1038/s41467-021-23282-x)
Supplement: Supplementary file 3 — Description of Additional Supplementary Files [file 41467_2021_23282_MOESM3_ESM.pdf]

Description of Additional Supplementary Files

File Name: Supplementary Software 1
Description: This folder contains three folders, one folder named as 'Cost_minimization' is used to optimize the retrofitting of power plants by county for biomass co-firing with CCS, one folder named as 'Electricity_path' is used to estimate the capacity of electricity generation in China over 2011-2030, and the last folder named as 'result' is used to store the generated results.

File Name: Supplementary Data 1
Description: Data for biomass feedstocks, gross domestic product (GDP), capacities of the electricity generation by power plants in 2015, and capacity for carbon storage are provided in this file.
