# Supplementary material for: Spatially explicit analysis identifies significant potential for bioenergy with carbon capture and storage in China
Source: Nat Commun. 2021 May 26;12:3159. doi: 10.1038/s41467-021-23282-x (PMC8154910; doi:10.1038/s41467-021-23282-x)
Supplement: Supplementary file 5 — Supplementary Software 1 [file 41467_2021_23282_MOESM5_ESM.zip › supplementary software 1/cover of supplementary software 1.docx]

Description:

This folder contains three folders, One folder named as ‘Cost_minimization’ is used to optimize the retrofitting of power plants by county for biomass co-firing with CCS, one folder named as ‘Electricity_path’ is used to estimate the capacity of electricity generation in China over 2011-2030, and the last folder named as ‘result’ is used to store the generated results.
